# Super-Tough and Environmentally Stable Aramid. Nanofiber@MXene Coaxial Fibers with Outstanding Electromagnetic Interference Shielding Efficiency

**DOI:** 10.1007/s40820-022-00853-1

**Published:** 2022-04-24

**Authors:** Liu-Xin Liu, Wei Chen, Hao-Bin Zhang, Lvxuan Ye, Zhenguo Wang, Yu Zhang, Peng Min, Zhong-Zhen Yu

**Affiliations:** 1 State Key Laboratory of Organic-Inorganic Composites, College of Materials Science and Engineering, Beijing University of Chemical Technology, Beijing, 100029 People’s Republic of China; 2 Beijing Key Laboratory of Advanced Functional Polymer Composites, Beijing University of Chemical Technology, Beijing, 100029 People’s Republic of China; 3 Beijing Advanced Innovation Center for Soft Matter Science and Engineering, Beijing University of Chemical Technology, Beijing, 100029 People’s Republic of China

**Keywords:** Core–shell fibers, MXene sheets, Electromagnetic interference shielding, Aramid nanofibers, Super-toughness

## Abstract

Although electrically conductive and hydrophilic MXene sheets are promising for multifunctional fibers and electronic textiles, it is still a challenge to simultaneously enhance both conductivity and mechanical properties of MXene fibers because of the high rigidity of MXene sheets and insufficient inter-sheet interactions. Herein, we demonstrate a core–shell wet-spinning methodology for fabricating highly conductive, super-tough, ultra-strong, and environmentally stable Ti_3_C_2_T_*x*_ MXene-based core–shell fibers with conductive MXene cores and tough aramid nanofiber (ANF) shells. The highly orientated and low-defect structure endows the ANF@MXene core–shell fiber with super-toughness of ~ 48.1 MJ m^−3^, high strength of ~ 502.9 MPa, and high conductivity of ~ 3.0 × 10^5^ S m^−1^. The super-tough and conductive ANF@MXene fibers can be woven into textiles, exhibiting an excellent electromagnetic interference (EMI) shielding efficiency of 83.4 dB at a small thickness of 213 μm. Importantly, the protection of the ANF shells provides the fibers with satisfactory cyclic stability under dynamic stretching and bending, and excellent resistance to acid, alkali, seawater, cryogenic and high temperatures, and fire. The oxidation resistance of the fibers is demonstrated by their well-maintained EMI shielding performances. The multifunctional core–shell fibers would be highly promising in the fields of EMI shielding textiles, wearable electronics and aerospace.

**Supplementary Information:**

The online version contains supplementary material available at 10.1007/s40820-022-00853-1.

## Highlights

- Super-tough and ultra-strong ANF@MXene fibers are wet-spun by a coaxial technique.
- High toughness of ~ 48.1 MJ m^−3^ and strength of ~ 502.9 MPa are achieved.
- The fibers exhibit superb chemical stability under extreme environmental conditions.

**Supplementary Information:**

The online version contains supplementary material available at 10.1007/s40820-022-00853-1.

## Graphical abstract

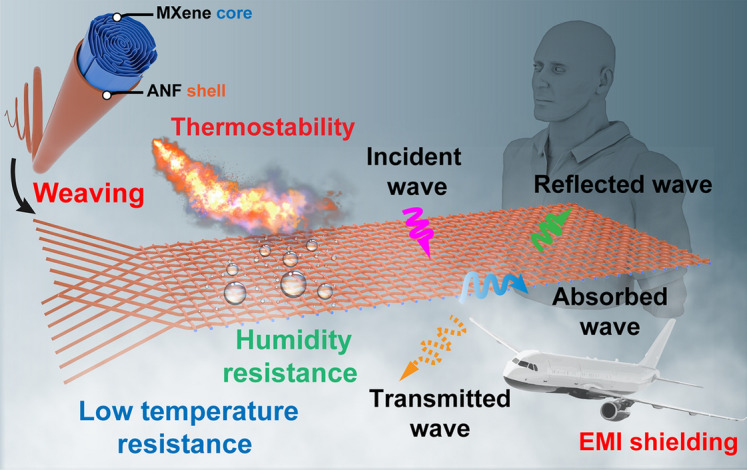

## Introduction

Electrically conductive, mechanically strong, and lightweight fibers with resistance to extreme conditions are urgently required for flexible and wearable electronics, aerospace, outer space and polar regions [1, 2]. As promising alternatives to traditional metal-based materials, various conductive carbon nanomaterials, including carbon nanotubes (CNTs) [3], graphene sheets [4] and transition metal carbides/nitrides (MXene) sheets [5], are explored for producing conductive fibers. Although the high electrical conductivity and favorable hydrophilicity features make the two-dimensional (2D) MXene sheets promising for electromagnetic interference (EMI) shielding, Joule heating, and functional textile applications [6–9], pristine MXene dispersion has a poor spinnability and its fibers are poor in tensile strength and ductility because of the weak inter-sheet interactions [10, 11]. Therefore, graphene oxide (GO) and/or polymers are introduced to improve the spinnability of the MXene spinning dopes [10, 12, 13]. With the presence of chemically reduced graphene oxide (RGO), the MXene-based fibers can exhibit an electrical conductivity of 2.8 × 10^3^ S m^−1^ and a tensile strength of 145.2 MPa [10]. Similarly, MXene/cellulose hybrid fibers show a tensile strength of 75.6 MPa and a conductivity of 211 S m^−1^ [14]. By adding a conductive polymer [15] or forming a solid coating layer [16], the conductivities of the MXene-based fibers could reach 1.49 × 10^5^ and 1.20 × 10^5^ S m^−1^, respectively, but their mechanical properties are less satisfactory. The increases in mechanical properties of MXene-based fibers are usually at the expense of their conductivities. Recently, Gogotsi and coworkers [11] reported the transition from isotropic phase to nematic phase by optimizing MXene size and spinning parameters, and the resultant wet-spun pristine MXene fibers exhibited a high electrical conductivity of ∼7.75 × 10^5^ S m^−1^. Similar high conductivities of 7.71 × 10^5^ and 7.20 × 10^5^ S m^−1^ were also achieved for pristine MXene fibers by cross-linking large MXene sheets with ammonium ions [17] or enhancing orientation of the sheets [18].

Currently, the oxidative degradation of MXene severely impedes practical applications of MXene fibers under complicated humid, thermal, and even extreme environmental conditions [19]. Additionally, the tensile strength and toughness of the pristine MXene fibers are unsatisfactory. Although coaxial spinning is a feasible approach for balancing mechanical performances and functionalities of MXene-based fibers by separately controlling their different components, the coaxial wet spinning of MXene fibers is at its early stage [5]. Recently, Li et al. [20] reported a strong MXene@GO core–shell fiber with 50 wt% of GO as the supporting shell, achieving a high strength of 290 MPa and an electrical conductivity of 2.4 × 10^3^ S m^−1^. Liu et al. [21] fabricated a hollow core–shell MXene fiber with a conductivity of 2.37 × 10^3^ S m^−1^ and a high toughness of 14.1 MJ m^−3^ by coaxial wet spinning of tough regenerated cellulose component and conductive MXene/GO component. Despite these encouraging advances, it still remains a great challenge to simultaneously enhance tensile strength, toughness, conductivity, and environmental stability of MXene fibers.

Herein, a coaxial wet-spinning strategy is adopted to fabricate super-tough, ultra-strong, environmentally stable, and highly conductive core–shell aramid nanofiber@MXene (ANF@M) fibers with the Ti_3_C_2_T_*x*_ MXene core and the aramid nanofiber (ANF) shell. The non-flammable and stable ANF shell plays crucial roles in improving the wet-spinnability of the MXene dopes and enhancing the mechanical performances and environmental stability of the MXene fibers. The resultant ANF@M fibers achieve a super-toughness of ~ 48.1 MJ m^−3^, a record-high tensile strength of ~ 502.9 MPa, and an outstanding conductivity of ~ 3.0 × 10^5^ S m^−1^. The excellent comprehensive performances of the ANF@M fibers allow them to be woven into functional textiles with an EMI shielding efficiency (SE) of up to 83.4 dB. The highly orientated MXene core and the strong ANF shell endow the highly conductive ANF@MXene fibers with satisfactory cyclic stability under dynamic stretching and bending, and outstanding resistance to oxidation. The influences of stretching ratio, shell thickness, concentration of the spinning dopes on mechanical and electrical properties of the core–shell fibers are explored. The resistances of the fibers to humidity, acid, alkali, seawater, cryogenic and high temperatures, and fire are also demonstrated.

## Experimental Section

### Materials

Ti_3_AlC_2_ powders (400 mesh) were purchased from the Jilin 11 technology. Lithium fluoride (LiF) and potassium hydroxide (KOH) were supplied by Aladdin (China). Hydrochloric acid (12 M) and ammonium chloride were bought from the Beijing Chemical Reagents and the Tianjin Guangfu Technology, respectively. Kevlar 49 fiber (PPTA) was provided by the DuPont. Dimethyl sulfoxide (DMSO) was obtained from Tianjin Damao Chemical Reagents (China).

### Preparation of MXene and ANF Spinning Dopes

Ti_3_C_2_T_*x*_ MXene was synthesized using a method reported elsewhere [22–25]. In a brief procedure, LiF (8 g) and HCl (9 M, 100 mL) were mixed in a Teflon vessel at a low temperature. After 5 g of Ti_3_AlC_2_ powder was added slowly, the resulting mixture reacted at 35 °C under stirring for 40 h. Subsequently, the resultant was washed with deionized water and ultrasonicated in an argon atmosphere for 1 h to obtain exfoliated MXene sheets. Finally, an aqueous suspension of MXene with a concentration of 50 mg mL^−1^ was prepared as a spinning dope. The MXene suspensions with concentrations of 50 and 20 mg mL^−1^ were designated as M50 and M20, respectively. Besides, for the synthesis of ANF dispersion, PPTA (1 g) and KOH (1 g) were added into the mixture of DMSO (100 mL) and deionized water (0.5 mL) and stirred magnetically for 1 week [26]. The resultant ANF dispersion was centrifuged at 3000 rpm for 30 min to remove impurities, obtaining a dark-red ANF dope (1 wt%) for wet spinning.

### Coaxial Wet Spinning of ANF@M Core–Shell Fibers

Core–shell ANF@M fibers were prepared by a coaxial wet spinning. The coaxial inner needle is 22 gauge (G, inner diameter ≈ 0.40 mm). The outer needles can be 18 G (inner diameter ≈ 0.85 mm), 17 G (inner diameter ≈ 0.95 mm), and 16 G (inner diameter ≈ 1.15 mm). At a constant stretching ratio (*R*) of 1.1, the influence of the thickness of the ANF shell is explored, and the as-prepared ANF@M fibers are designated as 18–1.1, 17–1.1, and 16–1.1 fibers. Unless otherwise specified, the concentration of the core layer MXene spinning dope was 50 mg mL^−1^. 17–1.1 and 17–1.1-50 M refer to the same fibers. 17–1.1-20 M means that the concentration of MXene core spinning dope was 20 mg mL^−1^. The concentration of the ANF spinning dope was fixed at 1 wt%. The MXene and ANF spinning dopes are injected, respectively, into the inner and outer needles of the coaxial needles by separate syringe pumps. The injection speeds of the pumps connected with the inner (22 G) and outer needles (17 G) are 150 and 400 μL min^−1^, respectively. When the coaxial outer needles were 18 G and 16 G, the injection speed of the inner needle was kept at 150 μL min^−1^, while the injection speeds of the outer needles were 218 and 780 μL min^−1^, respectively. Subsequently, the spun ANF@M fibers were coagulated in an aqueous coagulation bath with 5 wt% of ammonium chloride (NH_4_Cl), washed three times in ethanol/water (1:3, v/v) solvents, collected on reels, and dried naturally.

### Characterizations

Morphologies of MXene and ANF spinning dopes and fibers were observed using a Hitachi S4700 field-emission scanning electron microscope (SEM) and a Tecnai G2 F20 STWIN transmission electron microscope (TEM). Cross-sectional area of fibers was estimated using an ImageJ software on the basis of the cross-sectional SEM images. The birefringence of ANF@M fibers was observed using a Nikon Eclipse 80i polarized optical microscope (POM). SAXS and WAXS measurements were conducted on a Xenocs XEUSS 2.0 SAXS system equipped with a Genix^3D^ X-ray beam delivery at 50 kV and 0.6 mA, and a Thesea Pilatus3 R 200 K-A detector. The SAXS detector was placed at 2488 mm from the samples, while the WAXS detector was placed at 267 mm from the samples. The Herman’s orientation parameter (*f*) and orientation degree (*Π*) are quantified using Eqs. 1–3 [11, 27, 28]:

1 $$f = \frac{{3 < \cos ^{2} \varphi > - 1}}{2}{\text{ }}$$

2 $$< \cos^{2} \varphi > = \frac{{\mathop \smallint \nolimits_{ - \pi /2}^{\pi /2} I\left( \varphi \right) \sin \varphi \cos^{2} \varphi d\varphi }}{{\mathop \smallint \nolimits_{ - \pi /2}^{\pi /2} I\left( \varphi \right) \sin \varphi d\varphi }}$$

3 $$\Pi = { }\frac{{180 - {\text{fwhm}}}}{180}$$

where *φ*, *I*, and fwhm represent the azimuthal angle, the corresponding integral intensity, and the full width at half-maximum, respectively. The rheological properties of the aqueous suspensions of MXene and the ANF/DMSO solution were investigated using an Anton Paar MCR 102 Rheometer with a measuring plate CP-25 (25 mm). The shear rate varied from 0.01 and 1000 s^−1^. The viscoelastic properties were investigated by measuring the elastic modulus (G’) and viscous modulus (G’’) as a function of frequency from 0.1 to 100 Hz. During the frequency sweep, the strain amplitude was maintained at 0.1% (25 °C, a gap of 1 mm) [11, 17]. Mechanical properties of fibers were measured on a SUNS UTM4502XH tensile tester equipped with a load cell of 20 N at a cross-head speed of 1 mm min^−1^. Resistivities of the fibers with a length of 15 mm were evaluated with a two-probe method using a FLUKE 12E + multimeter. The electrical conductivity (σ) of the fibers was calculated using Eq. 4:

4 $$\sigma = L/\left( {RA} \right)$$

where *R*, *L,* and *A* are the resistance, the length and the conductive path area of a fiber, respectively. The humidity resistances were characterized using the self-made measuring equipment reported previously [22]. The resistance changes were recorded on a Keithley DMM7510 multimeter. Thermal stability of the fibers was investigated using a TA Q50 thermogravimetric analyzer (TGA) with a heating rate of 10 °C min^−1^ under an air atmosphere. Fire resistance test of the fiber was evaluated by burning the fibers in the outer flame of an alcohol lamp. MXene, ANF, and ANF@M fibers were characterized with a Thermo Fisher Nicolet 6700 Fourier-transform infrared (FTIR) spectrometer, and a ThermoVG RSCAKAB 250X X-ray photoelectron spectroscopy (XPS). EMI shielding performances were measured using a waveguide method on a Keysight N5224B PNA network analyzer within 8.2–12.4 GHz.

## Results and Discussion

### Coaxial Wet Spinning of ANF@MXene Core–Shell Fibers

**Fig. 1:**
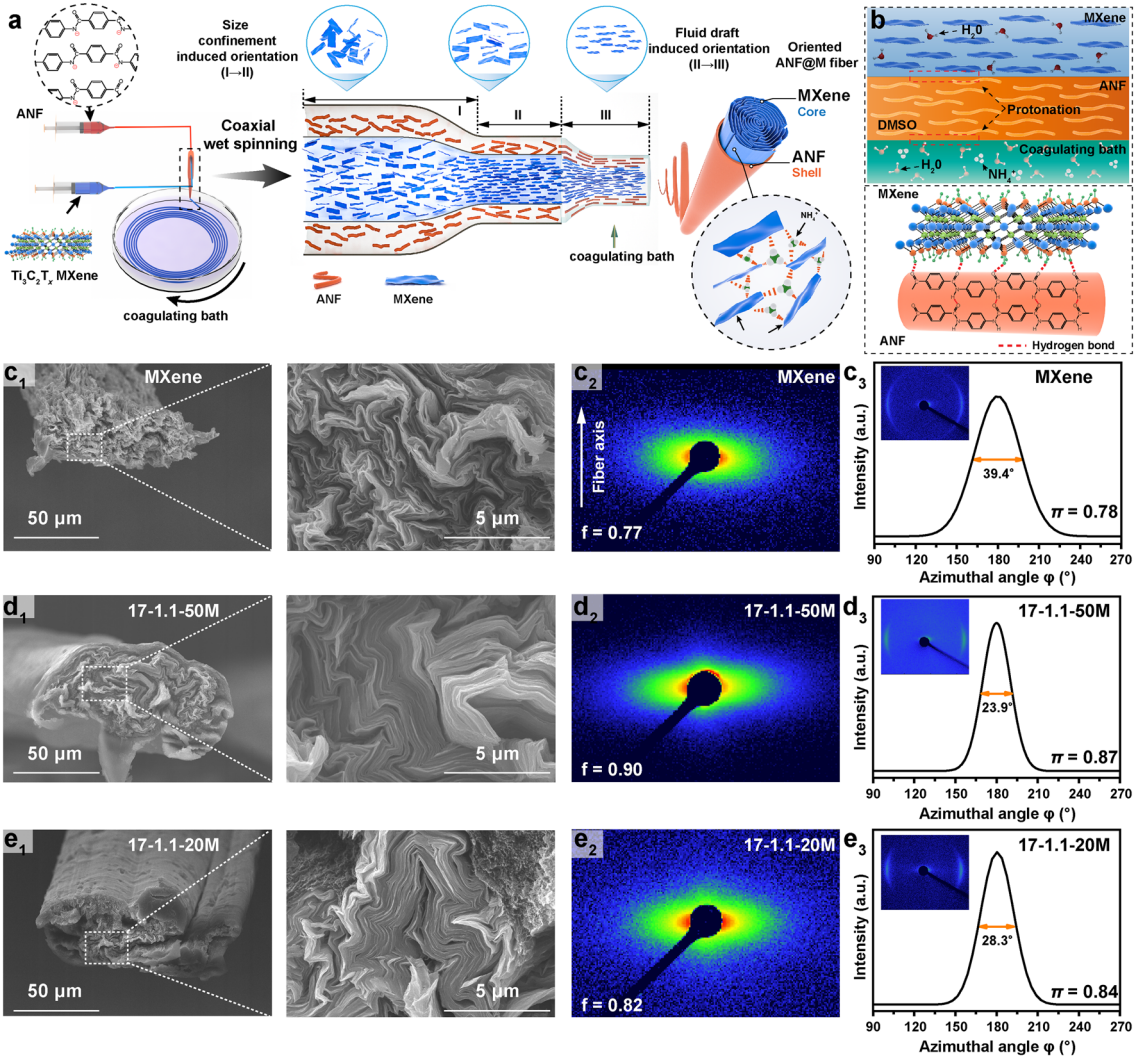
**a** Schematic illustrating the fabrication of core–shell ANF@M fiber by coaxial wet spinning, the evolution of the sheet orientation, and the cross-linking of MXene sheets with ammonium ions. **b** Protonation process of ANFs, and the interfacial interactions between MXene and ANF. **c**_**1**_, **d**_**1**_**, e**_**1**_ Cross-sectional SEM images, **c**_**2**_, **d**_**2**_**, e**_**2**_ 2D SAXS images, and **c**_**3**_, **d**_**3**_**, e**_**3**_ 2D WAXS images with corresponding *fwhm* curves for **c** MXene fiber, and **d, e** ANF@M fibers

To fully exploit the multifunctions of MXene, a coaxial wet-spinning technique is adopted to fabricate multifunctional core–shell ANF@M fibers with high performances and environmental stability by reinforcing the conductive MXene cores with the tough and non-flammable ANF shells (Fig. 1a). Firstly, a stable dark brown ANF/DMSO dispersion with numerous nanofibrils of ~ 11 nm in diameter and ~ 2 μm in length is prepared as a spinning dope by the deprotonation of poly(p-phenylene terephthalamide) (PPTA) fibers with KOH that can remove mobile H atoms from the amide groups of PPTA and weaken hydrogen bonding between PPTA chains (Figs. S1–S2) [26, 29]. Besides, a black aqueous suspension of Ti_3_C_2_T_*x*_ MXene sheets is obtained by etching MAX phase with HCl/LiF solution and subsequent ultrasonic exfoliation (Figs. S3–S4) [6, 22]. During the following wet-spinning process, the gradually narrowed spinning channel generates strong shear forces to align and orientate the outer aramid nanofibrils and the inner MXene sheets along the flowing direction. Subsequently, the spun fibers are coagulated in an aqueous solution of NH_4_Cl (5 wt%), where the NH_4_^+^ ions can effectively crosslink the negatively charged MXene sheets by electrostatic interactions [17, 30]. The aramid chains in the ANF shells are protonated by the water molecules from both the outer aqueous coagulation solution and the inner MXene aqueous suspension. Moreover, it can be seen from the FTIR spectra that the peak at 1645 cm^−1^ corresponding to the stretching vibration of the C=O for ANF shifts to 1630 cm^−1^ for the ANF@M, demonstrating the formation of hydrogen bonding between ANF and MXene (Fig. S5a). The binding energy of C–Ti–(OH)_x_ for MXene fiber reduces significantly for the ANF@M fiber characterized by the O 1* s* XPS spectra (Fig. S5b). The results indicate that the aramid chains can graft onto the MXene sheets by hydrogen bonding between the C=O groups of the ANF and the -OH groups of the MXene, enhancing the core–shell interfaces of the fibers (Fig. 1b). Therefore, the favorable coaxial wet-spinning strategy and the effective coagulation behavior ensure the formation of the compact and orientated structure, benefiting the enhancements in mechanical and electrical properties, and environmental stability.

For comparison, neat MXene fiber is prepared by uniaxial wet spinning of an aqueous MXene dispersion (50 mg mL^−1^) (Fig. S6), exhibiting a porous, rugged and wavy fiber surface with a relatively loose sheet stacking (Figs. 1c_1_ and S7a). In contrast, the ANF@M core–shell fiber has a distinct core–shell structure, smooth and uniform surface and well-stacked structure, which can be optimized by adjusting the experimental conditions, including the spinning parameters, and the contents of ANF and MXene components (Figs. 1d_1_-e_1_ and S7b). Generally, the ratio of the turntable rotation speed to the injection speed of the spinning dope from needle is defined as the stretch ratio (*R*), which is closely related to the structure and performances of the spun fibers (Fig. S8). At a low stretch ratio (*R* = 0.6), only freely crooked fibers with curved nodes are prepared, which do not show an apparent stretching effect (Fig. S8a-b). Interestingly, when the stretch ratio increases to 0.9 and above, uniform and smooth core–shell fibers are continuously spun (Fig. S8c-e). After the as-prepared wet ANF@M fiber is naturally dried at ambient conditions with its two ends fixed, its diameter becomes smaller with the removal of solvents and the improved orientation of the sheets (Fig. S9a-b). Figure S9c exhibits a typical ANF@M fiber with a length of 2 m.

Furthermore, small-angle X-ray scattering (SAXS) and wide-angle X-ray scattering (WAXS) are used to reveal the microstructures of the fibers by guiding the X-ray incidents perpendicular to the fiber axis (Figs. 1c–e and S10-S13). As expected, the fragile neat MXene fiber shows a lower Herman orientation factor (*f* = 0.77) and a less orientation (*Π* = 0.78) than all the core–shell ANF@M fibers (Fig. 1c) [18, 31]. It is worth noting that the neat MXene fiber is prepared using the inner needle with the same diameter as that used for the core–shell fibers. Also, the stretch ratio is 1.1 for preparing the neat MXene fibers. As reported, the orientation of the fibers depends highly on the stretch ratio, and the stacking manner of microfibrils and sheets can be optimized along the fiber axis [32, 33]. Clearly, the cross-sectional morphology of the fiber becomes waist-shaped, and the tightly arranged origami-like MXene core consists of compactly stacked MXene sheets (Figs. 1d and S11). Among the as-prepared core–shell ANF@M fibers, the highest *f* value (0.90) is achieved when the stretch ratio is 1.1, validating the largest microvoids alignment [30, 31]. Meanwhile, the ANF@M 17–1.1 fiber possesses the smallest half-maximum (*fwhm*) of 23.9° and the highest orientation degree of 0.87, demonstrating the best crystal plane orientation in the fibers. These results can be explained by the weak shearing effect on the sheets and microfibrils at the low stretch ratios and the incompatible orientation of the inner and outer layers at too high stretch ratios. Therefore, the obviously higher orientation degree of the ANF@M fibers than that of the MXene fiber highlights the crucial spatial confinement effect of the ANF shell on the stacking of the MXene core. The ANF shell is equivalent to a self-made confinement pipe during microfluidic spinning, and the inner MXene sheets are orientated along the confined space. Under the same stretch ratio, the orientation of the ANF@M fibers under the confinement of the ANF shell is better than that of neat MXene fibers [31].

Furthermore, the compositions of the ANF@M core–shell fibers are tuned by varying the diameter of the outer needle and the MXene concentration in its spinning dope. At the constant stretch ratio of 1.1, the larger outer needle yields core–shell fibers with thicker ANF shell and smoother outer surface, increasing the overall fiber diameter and altering the orientation degree of MXene sheets (Figs. 1e and S12). It is reasonable that a thin ANF shell would generate weak confinement on the inner MXene core and cause reduced orientation of MXene. For example, the 18–1.1 fiber shows lower *f* and *Π* values of 0.79 and 0.85 than those of the 17–1.1 fiber. However, a too-thick ANF shell also impacts the concentricity of the core–shell fiber during the wet-spinning process. Thus, the outer needle of 17 G is chosen to produce ANF@M fibers, and the MXene contents in the spinning dopes are 50 and 20 mg mL^−1^.

In view of the significant influence of the rheological behavior of a spinning dope on its spinnability, Fig. S14 compares viscosities and moduli of the spinning dopes of M20, M50, and ANF. The MXene dopes exhibit lower viscosity and modulus than those of ANF dope. Although the MXene dope with a MXene content of 20 mg mL^−1^ has a low viscosity, its *G’/G”* value is larger than 1, which is still suitable for wet spinning (Fig. S15) [11]. In particular, with the favorable confinement of the viscous ANF outer dope [31], the core–shell ANF@M fiber can still be spun continuously by using the spinning dope with 20 mg mL^−1^ of MXene. Compared to the neat MXene fiber prepared with the 50 mg mL^−1^ MXene dope, the core–shell ANF@M fibers of M20 and M50 possess much higher sheet alignment and orientation (Figs. 1c, e and S13). Therefore, the coaxial spinning with the ANF shell enhances the orientation and stacking of the MXene core and is well suitable for producing core–shell ANF@M fibers with outstanding mechanical and electrical properties and environmental stability.

### Mechanical Properties and Fracture Behavior of ANF@MXene Core–Shell Fibers

**Fig. 2:**
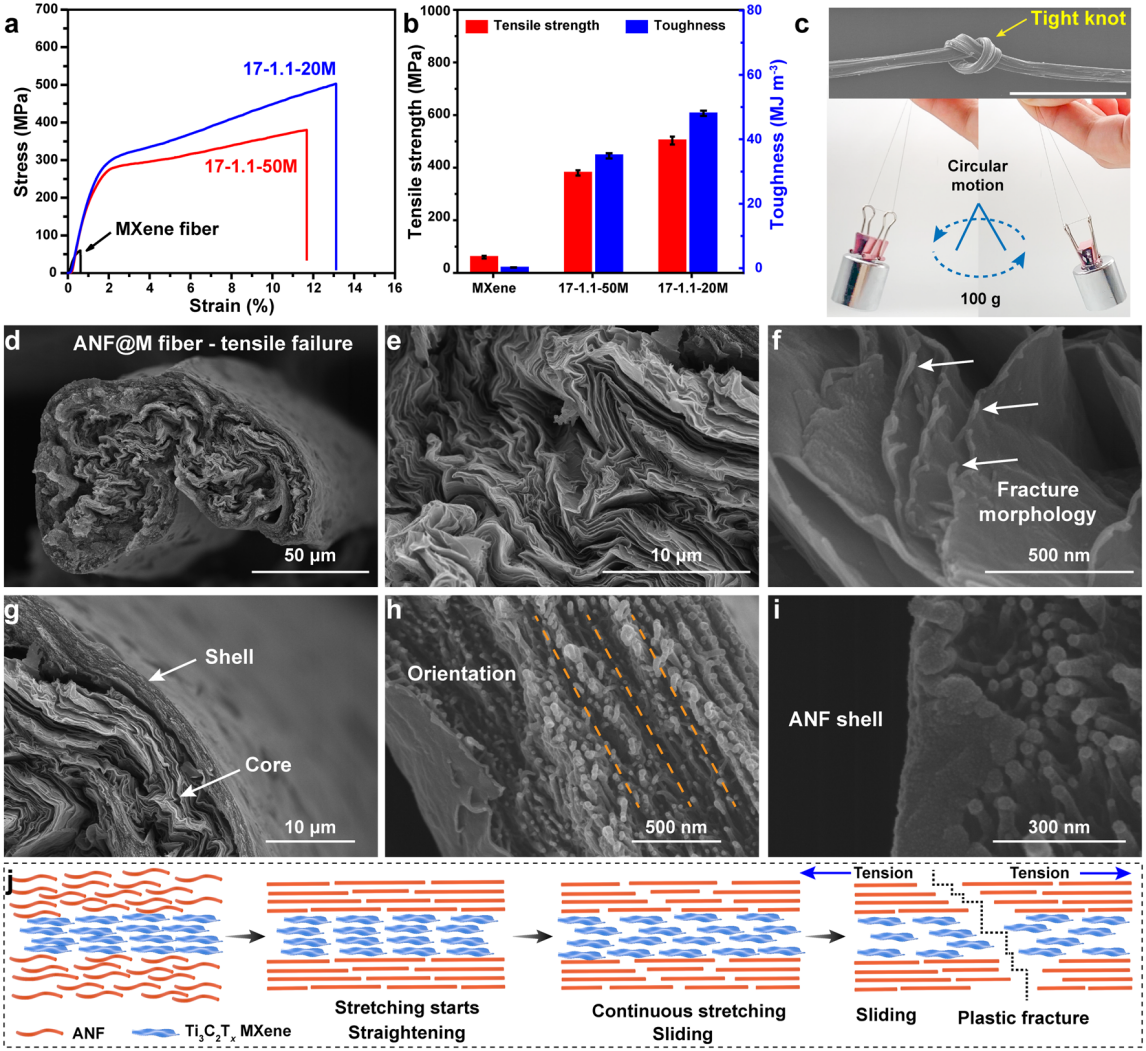
**a** Typical tensile stress–strain curves and **b** comparison of tensile strength and toughness of neat MXene fiber and ANF@M fibers. **c** SEM image of ANF@M fiber with a tight knot. An ANF@M fiber can support a weight of 100 g for circular motion. The scale bar is 1 mm. Cross section SEM images of **d, g** overall fracture surface, **e, f** MXene core, and **h, i** ANF shell of an ANF@M fiber. **j** Proposed fracture process of ANF@M fibers

To validate superiorities of the core–shell structure, Fig. 2a, b compares mechanical properties of neat MXene fiber and ANF@M fibers. As mentioned above, the spinning parameters of the neat MXene fiber are the same as those of the MXene component of the ANF@M core–shell fibers (*R* = 1.1). As expected, the fragile neat MXene fiber exhibits a low tensile strength of 59.9 MPa and a low toughness of 0.2 MJ m^−3^, which severely impedes its application. By contrast, the core–shell ANF@M fibers are much stronger and tougher with larger fracture strains. For example, the 17–0.9 core–shell fiber delivers a far high strength of 306.3 MPa, and an enhanced toughness of 29.3 MJ m^−3^ (Figs. S16, S17). Consistent with the fiber microstructures, the highest orientation degree endows the 17–1.1-50 M fiber with an even higher strength of 380.1 MPa, and a larger toughness of 34.9 MJ m^−3^ (Figs. 2a, b and S16, S17), which are 5.3 and 173.5 times higher than those of the neat MXene fiber, respectively. Moreover, the ANF shell thickness is crucial for the mechanical properties of the ANF@M fibers. For the 18–1.1 fiber with a low shell thickness, its tensile strength reaches 230.5 MPa with an elongation at break of 3.4% (Figs. S18, S19). Optimal comprehensive mechanical performances are obtained for the 17–1.1 fiber with a larger shell thickness [34]. Note that further increase in the shell thickness does not lead to further enhancement in mechanical properties, such as the 16–1.1 fiber, which may be attributed to the interface mismatch and the structural defects involved in the fibers with larger diameters [33]. It is seen that the diameters of the 18–1.1 and 17–1.1 fibers are similar, but the thickness of the ANF shell reduces almost by half (Table S3). Compared with the 17–1.1 fiber, the 16–1.1 fiber has a significant increase in the ANF shell thickness, but the diameter of the fibers also increases significantly. Therefore, the improvement in the mechanical properties of fibers should consider the effects of shell thickness and fiber diameter. When the fiber diameter is small, the influence of the ANF thickness is the main factor. When the shell thickness increases to a certain extent, the mechanical properties could not increase significantly due to the negative influence of the increase in fiber diameter.

The mechanical properties of the ANF@M fiber is optimized further by using the spinning dope with 20 mg mL^−1^ of MXene. Actually, the decrease in the MXene content directly reduces the volume of the MXene core and the diameter of the fiber, although the ANF shell thickness keeps unchanged. In accordance with the microstructures of the fibers (Figs. 1c-e and S13), the ANF@M fiber (17–1.1-20 M) fabricated with a spinning dope of 20 mg mL^−1^ MXene delivers an ultrahigh strength of 502.9 MPa, and a superb toughness of 48.1 MJ m^−3^, which are 32% and 38% higher than those of the 17–1.1-50 M fiber, respectively. To the best of our knowledge, the tensile strength and toughness of the 17–1.1-20 M fiber are record-high values for MXene-based fibers and films reported so far [5, 35]. The 17–1.1-20 M fiber is even stronger than commercial silver (230.6 MPa), copper (256.0 MPa) and aluminum (283.5 MPa) wires (Fig. S20, Table S1). For example, the tensile strength of the 17–1.1-20 M fiber is  ~ 96% higher than that of a copper wire, but the density of the former is only 27% of the latter. The lightweight, conductive, strong and tough fiber is promising for wearable electronics and electronic circuits. The mechanically strong and tough features enable a single ANF@M fiber to support a weight of 100 g for circular rotation (Video S1). The core–shell fiber can be freely folded, spirally twined on a glass rod, and even tied into a tight knot without fracture (Figs. 2c and S21). Its excellent fatigue resistance is also confirmed by the 150° bending–stretching tests for 5000 cycles (Fig. S22). To validate the stitchability, the fibers are woven into a multifunctional textile with a simple loom, which can withstand a weight of 200 g and vigorous bending and kneading (Figs. S23-S25, Video S2). The knitted fiber net can endure rapid impact of a table tennis descended from a large height (Fig. S26, Videos S3 and S4).

To analyze the contributions of different components, the theoretical strengths of the ANF@M fibers are calculated on the basis of the volume contents of the MXene core and the ANF shell and compared with the measured values of the ANF fibers (Table S2) [34]. The neat ANF fiber spun at *R* = 1.1 has an high tensile strength of 784.7 MPa (Figs. S8f and S27). Interestingly, the measured strength and modulus of the 17–1.1-50 M fiber are slightly higher than the theoretical values, giving a favorable synergistic effect between the ANF shell and the MXene core. In the case of 17–1.1-20 M fiber, the experimental and theoretical results reveal that the larger ANF volume content is the main reason for the significantly improved tensile strength and toughness, highlighting the dominant contribution of the ANF shell to the overall mechanical performances of the fibers.

To explore the fracture mechanisms, the cross-sectional surfaces of the ANF and ANF@M fiber after large-scale stretching are analyzed (Figs. 2d–i and S28–S30). Clearly, the typical core–shell structure is well retained without discernable interfacial detaching or separation, confirming the strong interactions between the MXene core and the ANF shell. Compared to the initial ordered feature of MXene layer, the rugged and rough MXene core reveals the severe pulling of sheets that occurred during the stretching process, and the curled sheet tips indicate a typical ductile fracture behavior (Fig. 2e, f). Similar to the fractured surface of neat ANF fiber, the solid shell shows numerous highly stretched and orientated microfibrils along the fiber axis (Figs. 2g–i and S28–S29). Based on these observations, a possible fracture mechanism of the ANF@M core–shell fiber is proposed (Fig. 2j). Initially, the stretching force straightens the curved ANF microfibrils and the crumpled MXene sheets, and their alignment and orientation extent are steadily enhanced with increasing the tensile strain. The tough ANF layer is largely elongated by stretching the microfibrils. However, the typical brittle fracture behavior of pristine MXene fibers is not observed for the MXene core, as clearly illustrated by the smooth stress–strain curve (Fig. 2a). Reasonably, a significant sheet-to-sheet sliding is expected for the MXene core to adapt the large strain, and the closely wrapped ANF shell provides constraint forces to ensure the steady sheet slippage rather than breakage. The strong interfacial interactions among the MXene core and the ANF shell can help effective load transfer and dissipate large energy for the core–shell fiber to fracture (Figs. 1a, 2a, j) [30, 35]. Therefore, the synergistic effects of the ANF shell and the MXene core, together with the high sheet orientation, compact stacking and strong interlayer interactions, are responsible for the extraordinary mechanical properties of the ANF@M fibers [36].

### Electrical and EMI Shielding Performances of ANF@MXene Core–Shell Fibers

**Fig. 3:**
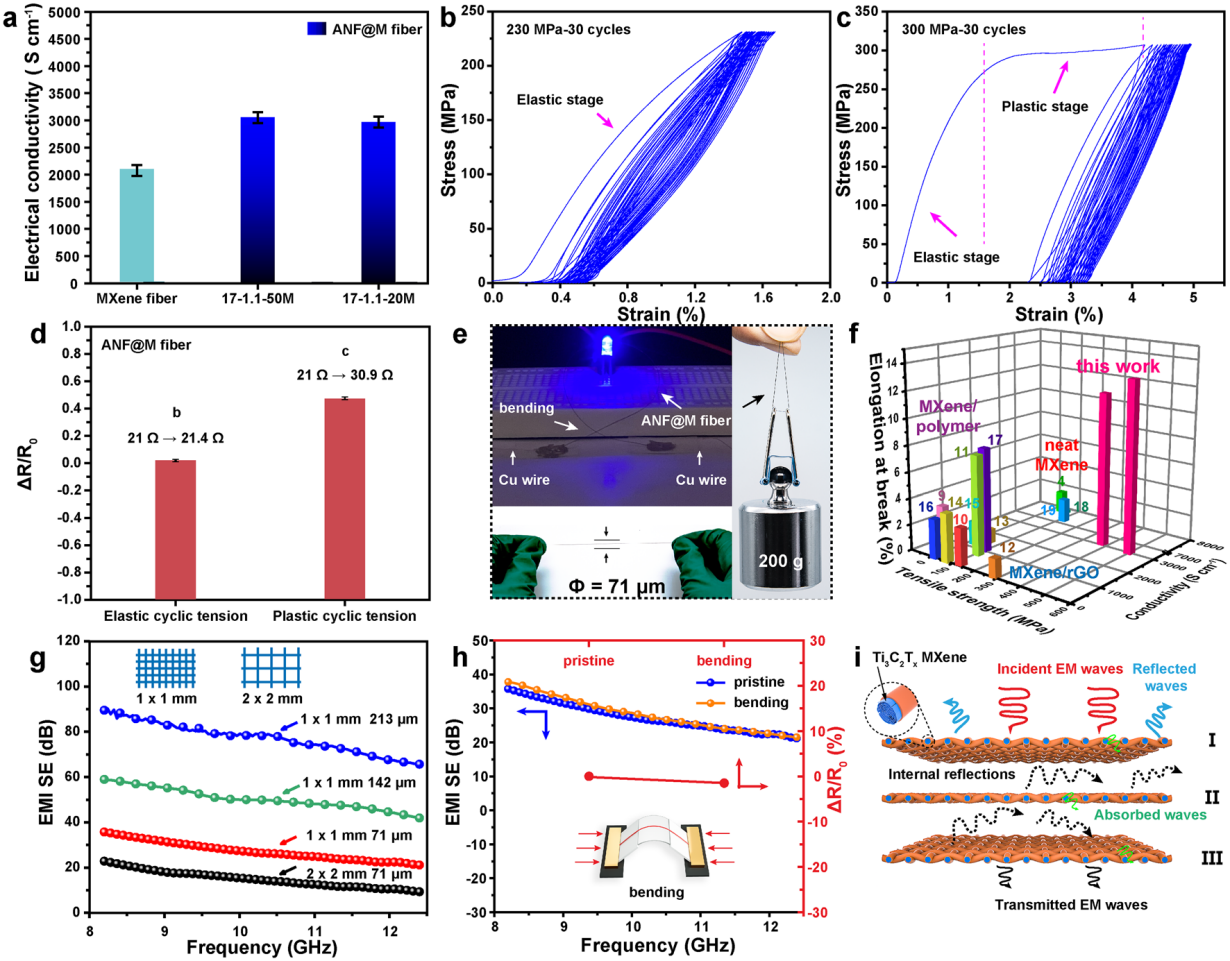
**a** Electrical conductivities of neat MXene fiber, and ANF@M fibers, and their cyclic tensile curves in **b** elastic stage and **c** plastic stage, and **d** corresponding resistance changes. **e** A 71-μm-thick conductive ANF@M fiber can bear a 200 g weight and light up a LED lamp under 1.8 V voltage with the length of 12 cm in a bent state. **f** Comparison of mechanical performances and conductivities of MXene-based fibers, and the numbers indicate the references listed in Table S3 in Supporting Information. **g** Plots of EMI SE versus different mesh grids and thicknesses of the 17–1.1-50 M textile. **h** Changes in resistance and EMI shielding performance after 5000 cycles of bending. **i** Possible EMI shielding mechanisms of the ANF@M textiles

In addition to the extraordinary mechanical performances, the core–shell ANF@M fibers are also featured by their high electrical and EMI shielding performances. As shown in Fig. 3a, the electrical conductivity of a loose neat MXene fiber is only 2.1 × 10^5^ S m^−1^, lower than those of all the strong and super-tough ANF@M fibers. For example, the ANF@M 17–1.1 and 18–1.1 fibers exhibit high conductivities of ~ 3.1 × 10^5^ and ~ 3.2 × 10^5^ S m^−1^, respectively, comparable to that (3.3 × 10^5^ S m^−1^) of the vacuum-filtrated MXene film (Fig. S31). Note that the ANF@M fiber with a low MXene content of 20 mg mL^−1^, which combines the best tensile strength and toughness also exhibits a superb electrical conductivity (~ 3.0 × 10^5^ S m^−1^). The conductivity of the ANF@M fibers can be enhanced further by using higher-quality MXene spinning dopes.

To satisfy the practical application requirements in wearable electronics [36, 37], the fatigue resistance of the ANF@M fiber is evaluated under cyclic stretching-release processes in the elastic and plastic stages, and its resistance variation is monitored (Fig. 3b, c). Clearly, the stress–strain curves show a good repeatability after the first elastic and plastic stretching, and corresponding changes in fiber resistances are very small, if not negligible (Fig. 3d). The structure robustness and the fatigue resistance of the ANF@M fiber ensure its potential applications. Typically, a 71-μm-thick ANF@M fiber can carry a weight of 200 g, and it can connect the LED lamp as a conductive wire even in a bent state (Fig. 3e). Clearly, the ANF@M fibers exhibit superior comprehensive properties over those of pristine MXene fibers [11, 17, 18], MXene/RGO fibers [10, 12, 20, 38], and MXene/polymer fibers [14–16, 39, 40] (Fig. 3f). The 17–1.1–20 M fiber combines a tensile strength of over fivefold that of the composite fibers and pristine MXene fibers, a record-high toughness, and a high conductivity comparable to pristine MXene fibers (Table S4).

The excellent comprehensive performances of the ANF@M fiber enable it to be woven into mesh/textiles for EMI shielding applications. The EMI shielding effectiveness (SE) depends highly on mesh spacing and thickness of the conductive layer. It can be tuned from 18.1 to 31.5 dB with decreasing the mesh spacing from 2 to 1 mm because of the improved capability of dissipating electromagnetic waves (Fig. 3g) [41]. Meanwhile, the increment in the textile thickness also enhances the shielding performance effectively to 55.4 (142 μm) and 83.4 dB (213 μm) at a constant mesh spacing of 1 mm. Additionally, consistent with the resistance changes, the EMI shielding performances of the ANF@M fiber remain stable even after bending to 150° for 5000 cycles (Fig. 3h), highlighting the much better cyclic stability and fatigue resistance than those of the fibers/textiles coated with active materials [42, 43]. The study on EMI shielding mechanism of the textile reveals that the small pore or multiple layers benefit the enhancement in overall EMI shielding performances by mainly increasing the absorption contribution (Figs. S32, S33) [7, 44]. The smaller the mesh spacing, the more perfect the conductive network, and the stronger the ability to reflect and dissipate electromagnetic waves [45, 46]. The thicker the textile, the larger the area where the electromagnetic waves are reflected, scattered and dissipated in the form of heat [47–49]. As illustrated in Fig. 3i, in addition to the incident electromagnetic waves reflected on the conductive textile surface, the incident waves entering into the interior of the textile can be dissipated by multiple scattering, abundant interfacial polarization, and conducting loss [50, 51].

### Stability of ANF@MXene Core–Shell Fibers under Extreme Environments

**Fig. 4:**
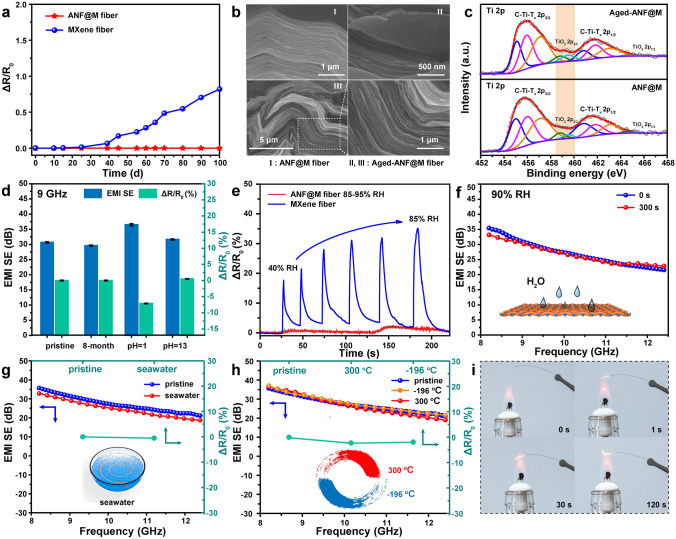
**a** Plots of the resistance change versus time for neat MXene fiber and ANF@M fiber. **b** SEM images of as-prepared ANF@M fiber (I) and after 8-month aging (II, III), and their **c** Ti 2p XPS spectra. **d** Resistance changes and shielding performances after aging for 8-month, and in acid and alkali solutions for 20 h. **e** Humidity-dependent resistance changes of neat MXene fiber and ANF@M fiber. EMI shielding performances of ANF@M fiber **f** under a relative humidity of 90%, and **g** in seawater. **h** Resistance changes of ANF@M fiber at different temperatures and shielding performances of the ANF@M fiber treated at different temperatures. **i** Non-flammability of the ANF@M fiber in fire

The ANF@M fibers possess exceptional long-term stability in a humid environment, at extreme temperatures, and in acid and alkali media. Under ambient conditions, neat MXene fiber begins to degrade after 25 days as indicated by the increased electrical resistances (Fig. 4a), and it is even collapsed by sonicating in water for 10 s (Fig. S34). By contrast, because of the ANF shell protection, the core–shell fiber is pretty stable even after 8 months or being sonicated for 120 s in water (Figs. 4b, c and S34). The stability of the ANF@M fiber can be validated by the stable electrical resistances and the invariable Ti 2*p* XPS spectra of the fiber after 8-month aging (Fig. 4c, d). Moreover, the high sensibility to humidity makes the MXene fiber shows a large resistance change of 35% at 85% relative humidity (Fig. 4e), similar to the MXene-decorated fabrics [22]. In contrast, the resistance change of the ANF@M fiber is as small as 3% even after the long-term stay under a relative humidity of 90% (Fig. S35). Accordingly, the EMI shielding performance of the prepared textile remains almost unchanged even after placing under a relative humidity of 90% for 300 s, or in seawater for 20 h (Fig. 4f, g). After soaking in harsh acid or alkali solutions for 20 h, the ANF@M fiber still exhibits excellent electrical and EMI shielding performances (Fig. 4d). Interestingly, the acid solution (pH = 1) can decrease the resistance and improve the EMI SE value of the ANF@M fiber from 31.5 to 37.4 dB. As reported, the acid treatment could enhance the interlayer interaction of MXene sheets and hence increase their electrical conductivity [52]. Whereas in an alkaline solution (pH = 13), the ANF@M fiber shows negligible variations in resistance change and EMI shielding performance (Fig. 4d). Thus, the ANF@M fiber can satisfy potential applications in extreme solvent environments.

As well known, the poor thermal stability of MXene often impedes potential applications under extreme temperature conditions. Fortunately, the ANF@M fiber-derived textile does not show apparent degradation in performances even after heating at 300 °C for 5 h in a tube furnace or cooling in liquid nitrogen (− 196 °C) for 1 h, affording remarkable cryogenic and high temperatures (Fig. 4h). In addition, the ANF@M fiber shows a good fire resistance and non-flammability in fire (Fig. 4i). For example, it does not burn even under the flame of an alcohol lamp, although MXene is oxidized to titanium dioxide under high temperature flame in an air environment. And the mass retention rate of the ANF@M fiber at 800 °C is up to 75.6%, due to the high temperature resistance of the ANF and the inorganic MXene (Fig. S36). All these results indicate that the lightweight, strong and super-tough ANF@M fibers with outstanding electrical conductivity and environmental stability are promising for applications in aircraft, polar workstations, wearable electronics, and artificial intelligence materials.

## Conclusions

Super-tough, highly conductive, ultra-strong, and environmentally stable core–shell fibers with the conductive MXene core and the tough ANF shell are fabricated by a core–shell spinning methodology. By optimizing the stretching ratio, the shell thickness, and the content of MXene in its spinning dope, the resultant ANF@M core-shell fiber exhibits a high conductivity of ~ 3.0 × 10^5^ S m^−1^, a super-toughness of ~ 48.1 MJ m^−3^, and a high tensile strength of ~ 502.9 MPa. The effective barrier effect of the ANF shell on the internal MXene core endows the ANF@M fibers with excellent resistances to acid, alkali, seawater, humidity, cryogenic and high temperatures, and tensile and bending fatigue. The super-tough and conductive fibers can be woven into textiles, exhibiting an excellent EMI SE of 83.4 dB at a small thickness of 213 μm. The outstanding mechanical and electrical properties along with the environmental stability make the MXene-based fibers promising in the areas of electromagnetic interference shielding, wearable electronics, wires, and aerospace.

## Supplementary Information

Below is the link to the electronic supplementary material.

Supplementary file1 (MP4 9285 KB)

Supplementary file2 (MP4 21771 KB)

Supplementary file3 (MP4 6194 KB)

Supplementary file4 (MP4 5483 KB)

Supplementary file5 (PDF 3036KB)
