# Supplementary material for: Super-Tough and Environmentally Stable Aramid. Nanofiber@MXene Coaxial Fibers with Outstanding Electromagnetic Interference Shielding Efficiency
Source: Nanomicro Lett. 2022 Apr 24;14:111. doi: 10.1007/s40820-022-00853-1 (PMC9035413; doi:10.1007/s40820-022-00853-1)
Supplement: Supplementary file 5 — Supplementary file5 (PDF 3036KB) [file 40820_2022_853_MOESM5_ESM.pdf]

Supporting Information for

Super-Tough and Environmentally Stable Aramid Nanofiber@MXene Coaxial Fibers with Outstanding Electromagnetic Interference Shielding Efficiency

Liu-Xin Liu[1, 2], Wei Chen[1], Hao-Bin Zhang[1, *], Lvxuan Ye[1], Zhenguo Wang[1], Yu Zhang[2], Peng Min[1], and Zhong-Zhen Yu[2, 3, *]

[1] State Key Laboratory of Organic-Inorganic Composites, College of Materials Science and Engineering, Beijing University of Chemical Technology, Beijing 100029, P. R. China

[2] Beijing Key Laboratory of Advanced Functional Polymer Composites, Beijing University of Chemical Technology, Beijing 100029, P. R. China

[3] Beijing Advanced Innovation Center for Soft Matter Science and Engineering, Beijing University of Chemical Technology, Beijing 100029, P. R. China

*Corresponding authors. E-mails: zhanghaobin@mail.buct.edu.cn (H.-B. Zhang); yuzz@mail.buct.edu.cn (Z.-Z. Yu)

Supplementary Figures and Tables

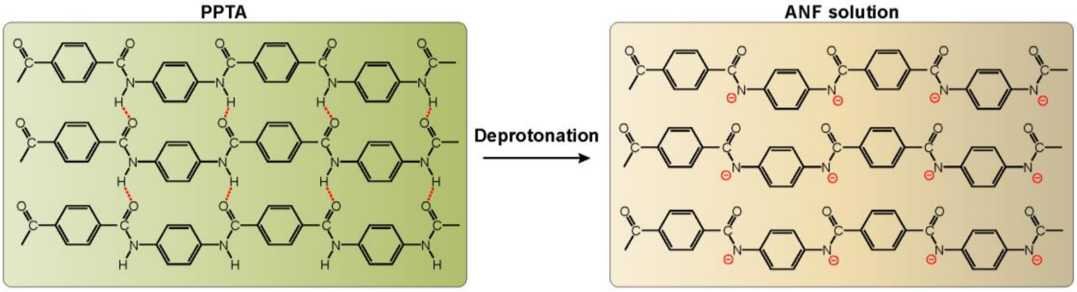

Fig. S1 Schematic illustrating the deprotonation of PPTA fibers with KOH to form an ANF solution

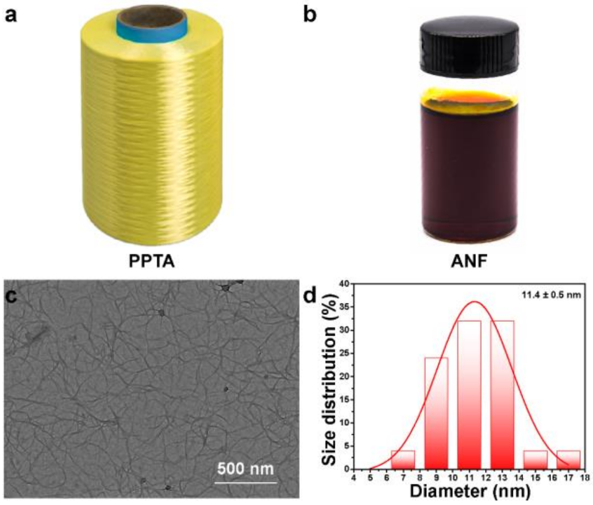

Fig. S2 Digital photos of **a** PPTA fibers and **b** an ANF dispersion. **c** TEM image and **d** size distribution of ANFs

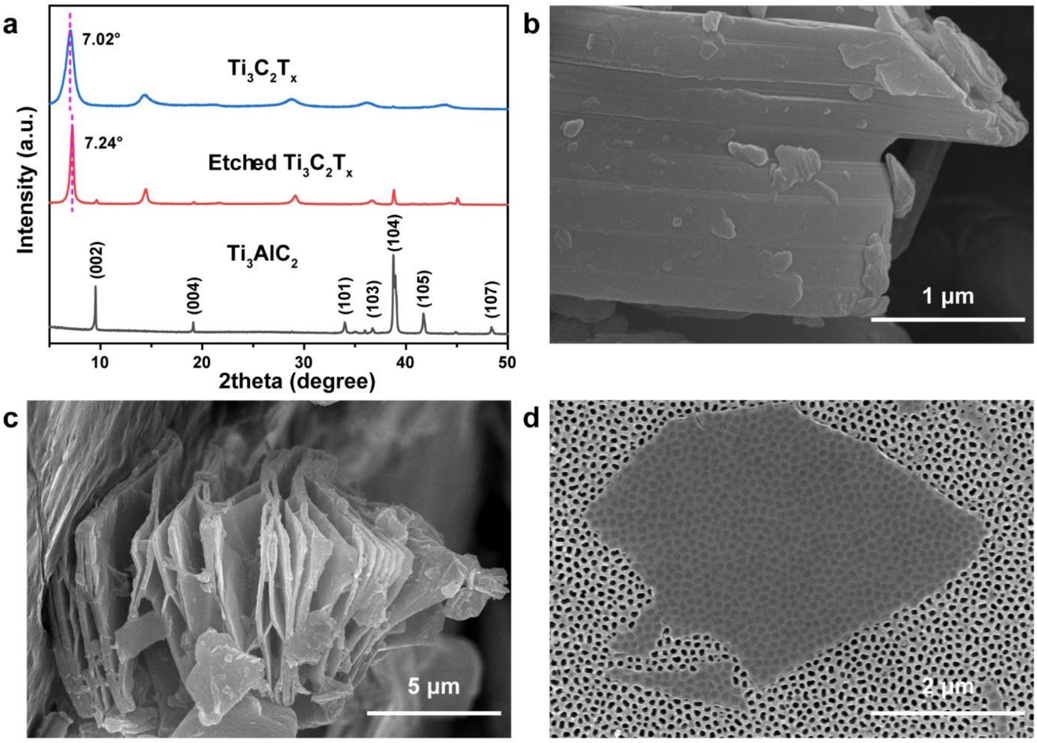

Fig. S3 a XRD patterns of Ti_3AlC_2 MAX phase, stacked $Ti_3C_2T_x$, and exfoliated $Ti_3C_2T_x$ sheets. SEM images of **b** Ti_3AlC_2 MAX phase, **c** stacked $Ti_3C_2T_x$, and **d** exfoliated $Ti_3C_2T_x$ sheets on a porous anodic aluminum oxide

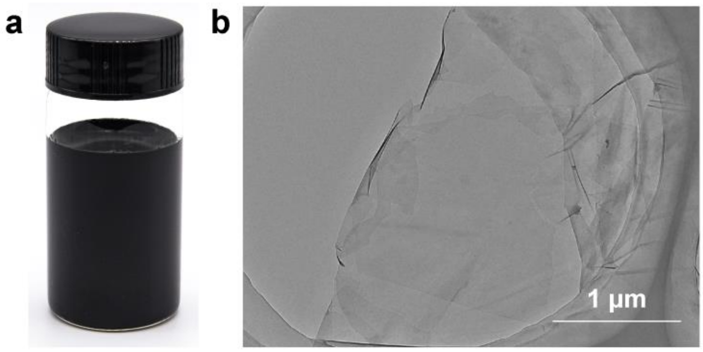

Fig. S4 a Digital photo and **b** TEM image of a MXene dispersion

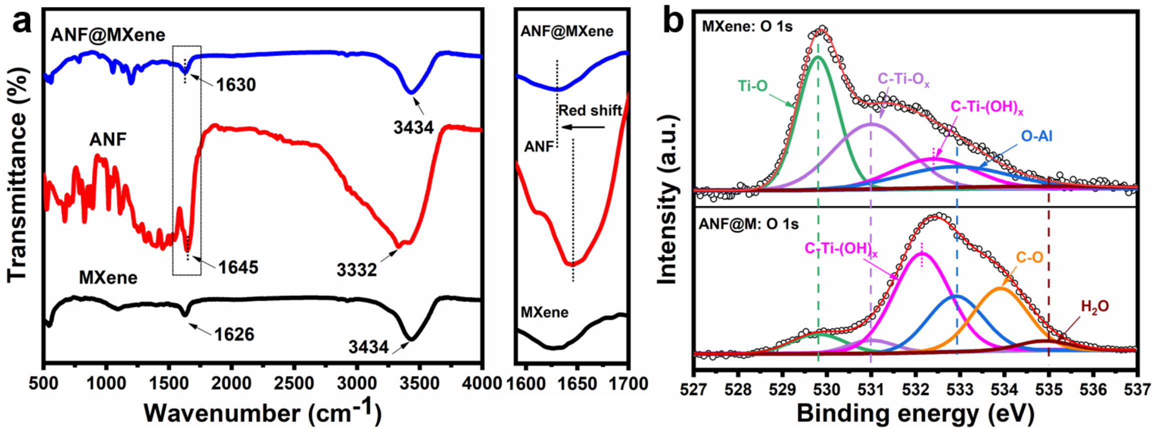

Fig. S5 a FTIR spectra of MXene, ANF, and ANF@M. **b** O 1s XPS spectra of MXene and ANF@M fiber

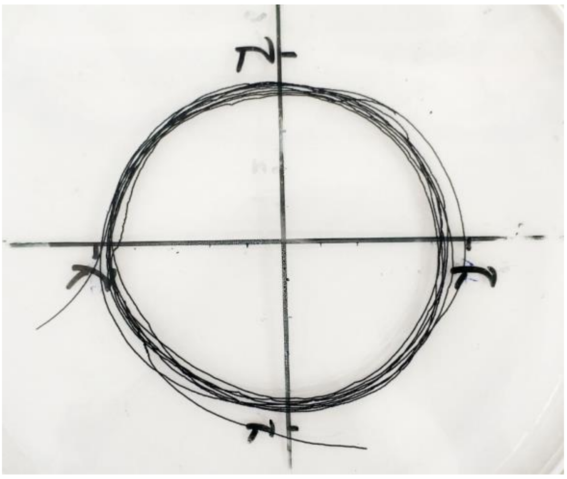

Fig. S6 Digital photo of neat MXene fiber

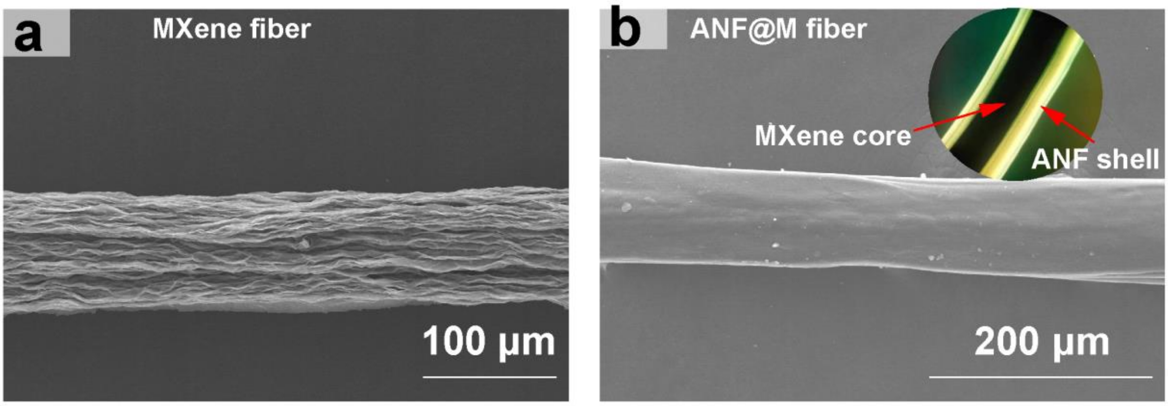

Fig. S7 SEM images of **a** neat MXene fiber, and **b** ANF@M fiber. The inset in **b** shows the core-shell structure of the ANF@M fiber characterized by a polarized optical microscopy

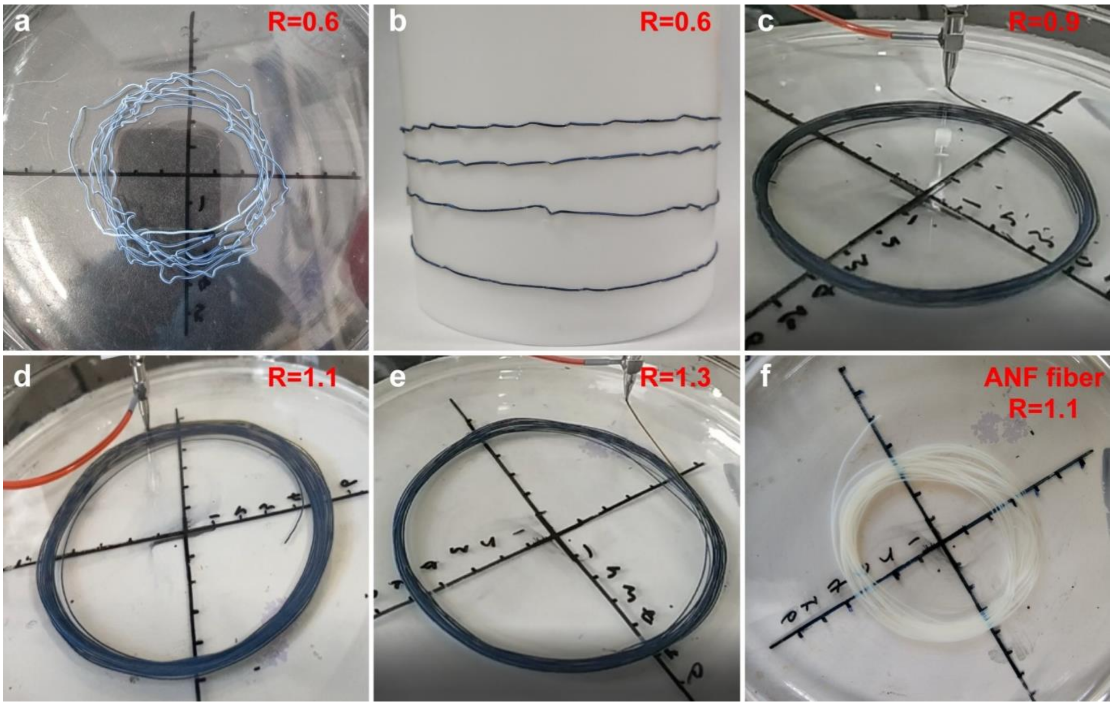

Fig. S8 Digital photos of **a-e** ANF@M fibers under different stretch ratios, and **f** pure ANF fiber

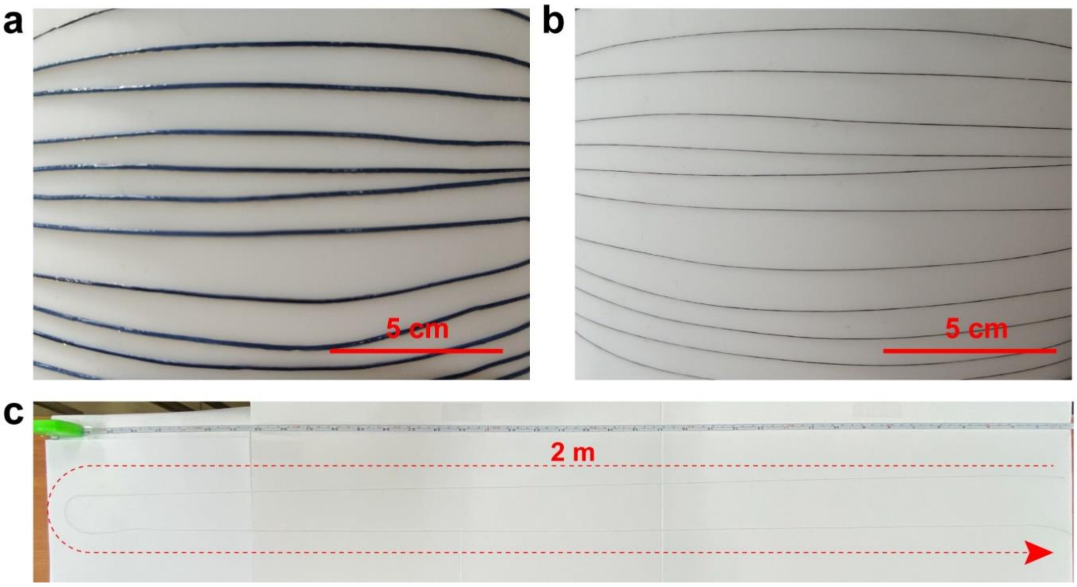

Fig. S9 a, b Shrinkage change of ANF@M fibers during drying. **c** Digital photo of a 2-meter long ANF@M fiber

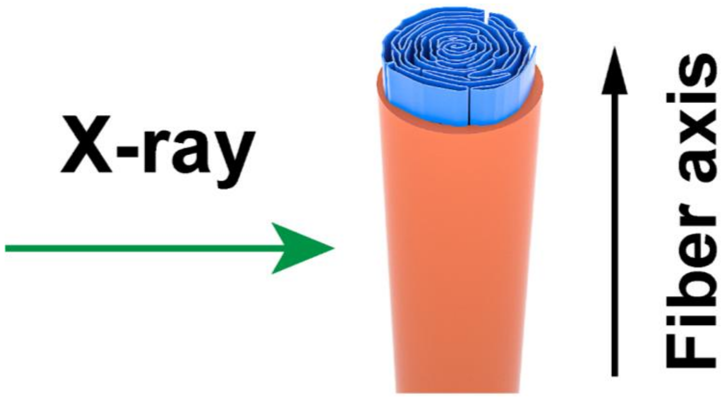

Fig. S10 Schematic diagram for small-angle x-ray scattering (SAXS) and wide-angle x-ray scattering (WAXS) measurements

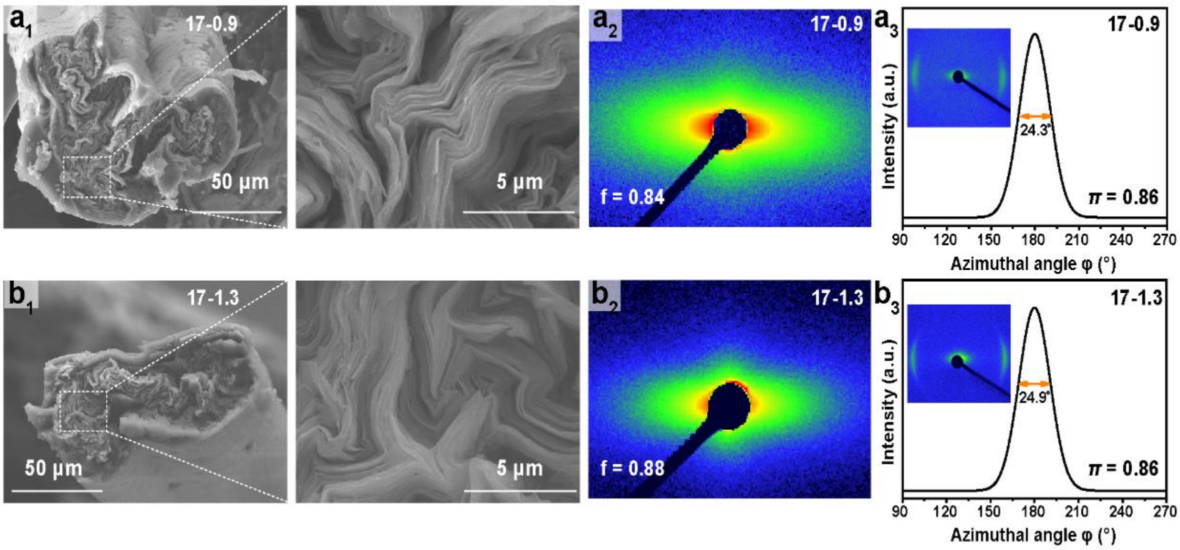

Fig. S11 a_1, b_1 SEM images, **a_2, b_2** 2D SAXS images, and **a_3, b_3** WAXS images with corresponding fwhm curves of the ANF@M fibers with different stretch ratios.

Differently, the *f* value of the 17-0.9 fiber can reach 0.84, and its *Π* value becomes 0.86 (**Fig. S11**). In particular, the ANF@M fibers have significantly higher orientation than pure MXene fibers, highlighting the spatial confinement effect of the ANF shell on the improvement of orientation and arrangement of the MXene core. In addition to the drawing effect, the fluid drafting also provides favorable confinement in radial fiber direction [S1]. With the increase of *R* value, the overall fiber structure steadily becomes waist-shaped, and the tightly arranged origami-like MXene core indicates a higher order arrangement of MXene sheets during the spinning process (**Figs. 1d and S11**). As reported, the orientation degree of the fibers depends on the *R*, and the appropriate *R* can maximize the order arrangement of the microfibrils and nanosheets along the fiber axis [S2, S3]. Interestingly, the 17-1.1 fiber shows the highest *f* value of 0.90 as compared to those of 17-0.9 (0.84) and 17-1.3 (0.88) fibers. Consistently, the 17-1.1 fiber has the smallest half-maximum (fwhm) of 23.9° and the highest orientation degree (*Π*) of 0.87, validating the largest crystal plane orientation [S1]. To explain these results, the weak tensile force at low *R* only cannot fully stretch the sheets and microfibrils; whereas, a too high *R* also causes incompatible orientation of the inner and outer layers due to their different rheological properties and fluidities

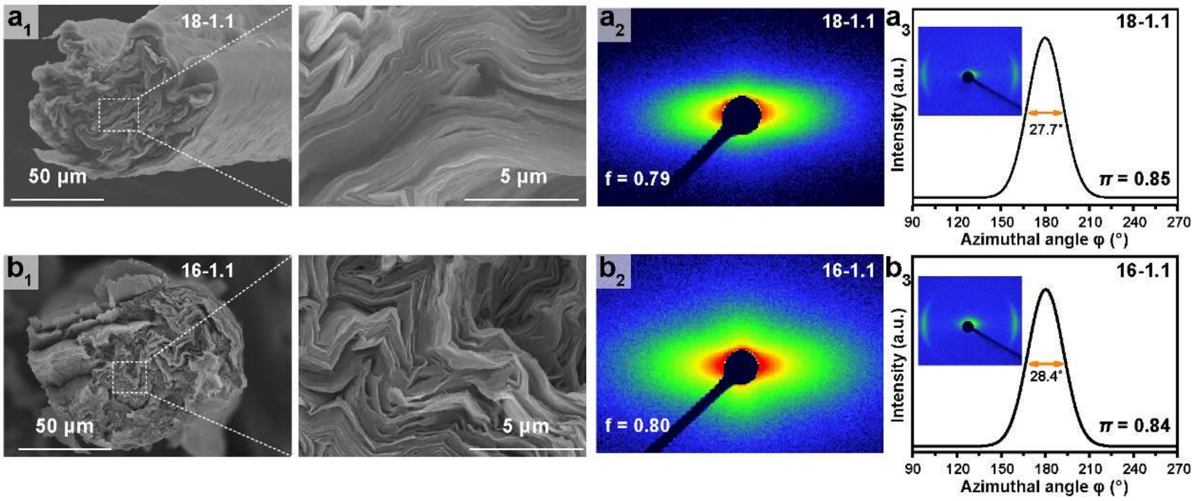

Fig. S12 **a$_1$, b$_1$** SEM images, **a$_2$, b$_2$** 2D SAXS images, and **a$_3$, b$_3$** WAXS images with corresponding fwhm curves of the ANF@M fibers with different shell thicknesses

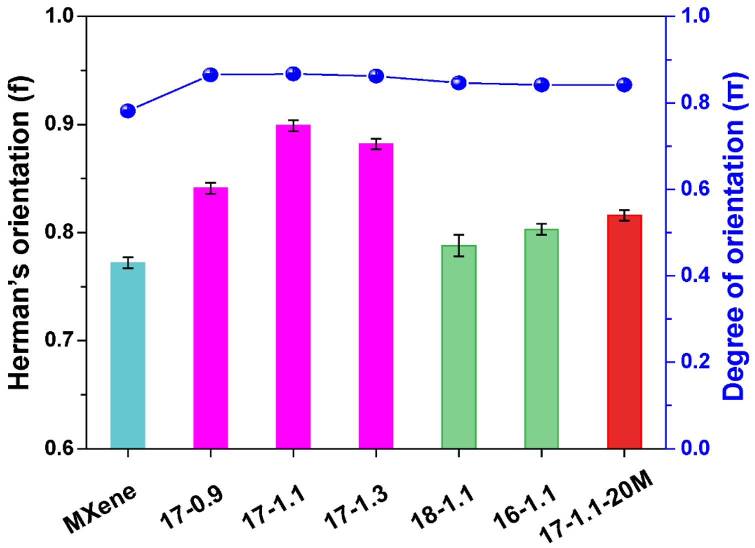

Fig. S13 Herman orientation factor (*f*) and orientation degree (*Π*) of the fibers

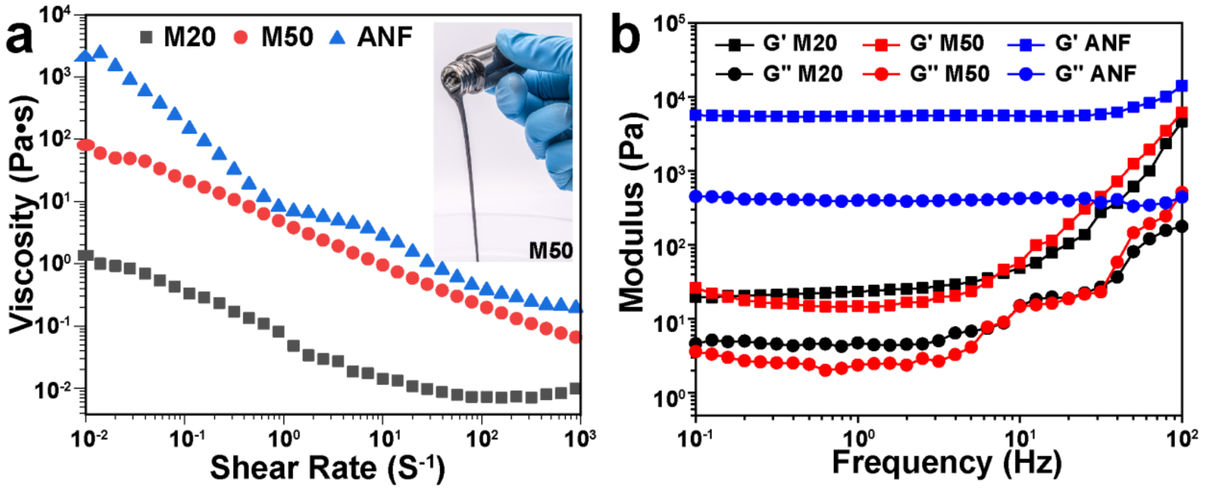

Fig. S14 a Plots of viscosity as a function of shear rate for M20, M50, and ANF spinning dopes. **b** Plots of G' and G" as a function of frequency for M20, M50 and ANF spinning dopes

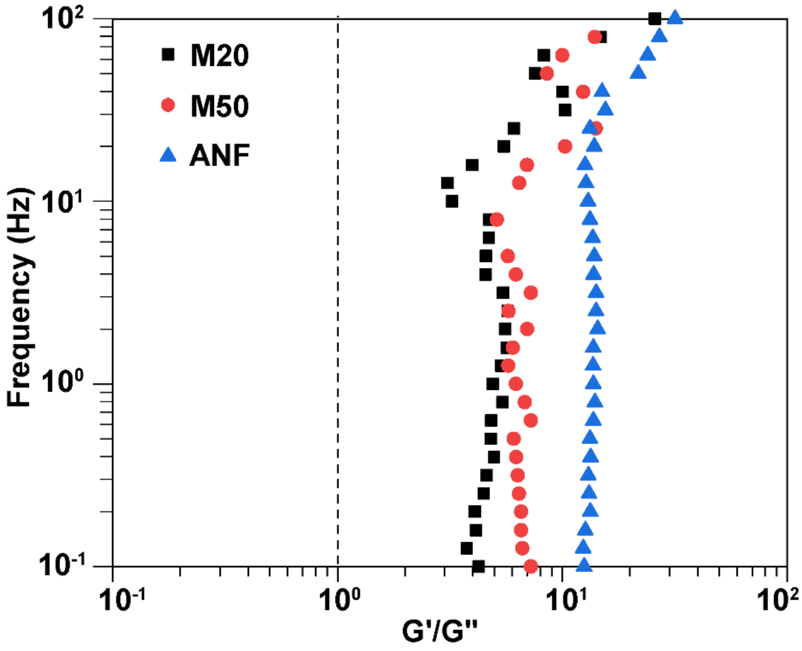

Fig. S15 Plots of frequency dependency on G′/G″ ratio for M20, M50, and ANF spinning dopes

The rheological properties of the spinning dopes are critical for the spinnability and fiber morphology. The zero-shear viscosity of the MXene suspension can reach 100 Pa·s at 50 mg mL^{-1} (M50) (**Fig. S14a**). The reduction of MXene content from 50 to 20 mg mL^{-1} (M20) obviously decreases the viscosity of the spinning dope. Note that the viscosity of the ANF dispersion is far higher than that of the MXene spinning dopes (M50, M20), as evidenced by the almost frequency-independent modulus within most frequency range explored (**Fig. S14b**). In contrast, the elastic modulus (*G'*) and viscous modulus (*G''*) of M20 and M50 are more dependent on the frequency. However, the *G'/G''* values of the three spinning dopes are all greater than 1. Their viscoelastic gel properties make them suitable for wet spinning (**Fig. S15**) [S4].

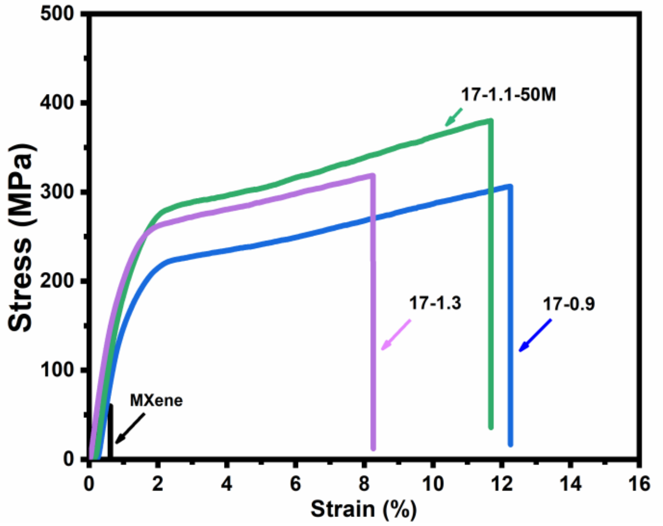

Fig. S16 Tensile stress–strain curves of ANF@M fibers under different stretch ratios, and neat MXene fiber

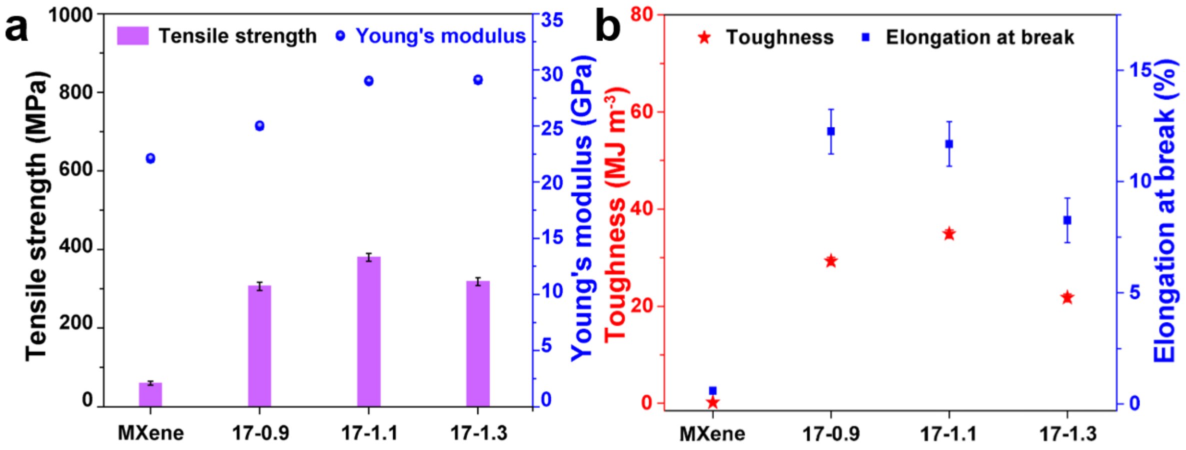

Fig. S17 Comparison of **a** tensile strength and Young's modulus, and **b** toughness and elongation at break of ANF@M fibers under different stretch ratios with those of neat MXene fiber

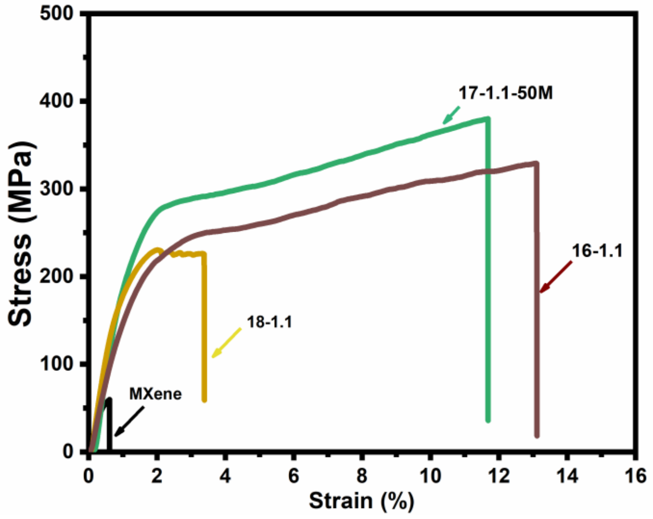

Fig. S18 Tensile stress–strain curves of ANF@M fibers under different shell thickness, and neat MXene fiber

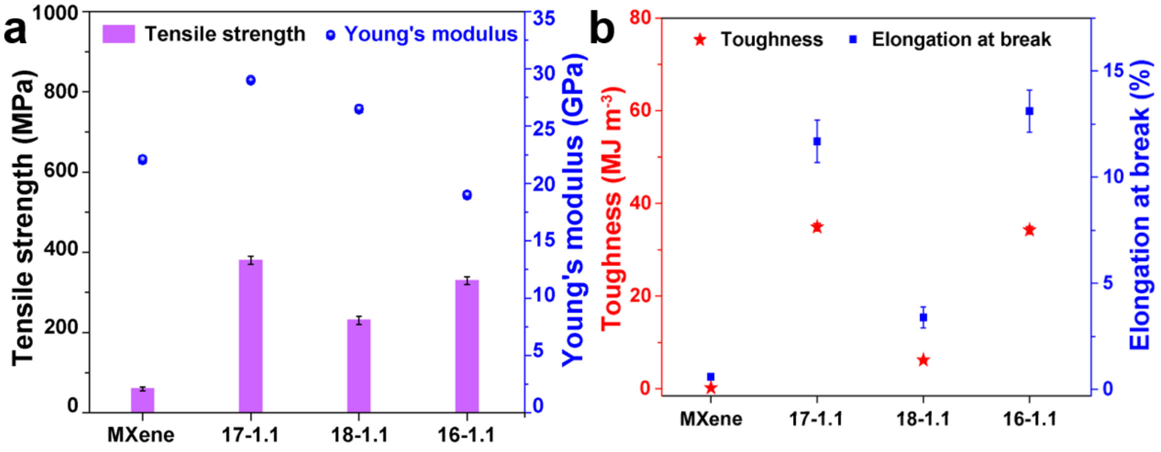

Fig. S19 Comparison of **a** tensile strength and Young's modulus, and **b** toughness and elongation at break of ANF@M fibers under different shell thicknesses with those of neat MXene fiber

The shell thickness also plays a crucial role in determining the mechanical properties of ANF@M fibers. When the shell thickness is reduced, the tensile strength and elongation at break are reduced to 230.5 MPa and 3.4% (18-1.1 fiber), respectively (**Fig. S18-S19**). The decrease in the thickness of the ANF shell results in a decrease in the comprehensive mechanical properties of the fiber, which proves the importance of the high-performance shell [S5]. However, when the shell thickness increases, the elongation at break of the 16-1.1 fiber are 13.1%, higher than that of 17-1.1 fiber; whereas the toughness and tensile strength of 16-1.1 fiber are 34.2 MJ m^{-3} and 329.2 MPa, similar to those of 17-1.1 fiber. The reason is that when the core layer is unchanged and the thickness of the shell layer is increased, the overall diameter of the fiber increases, and the interface defects also increase accordingly.

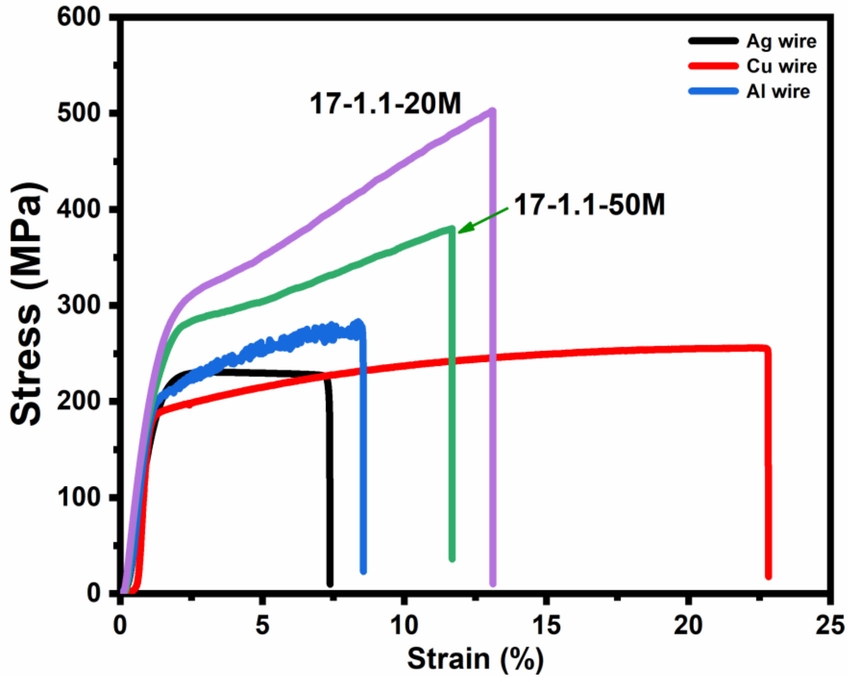

Fig. S20 Typical tensile stress–strain curve for Ag, Cu, Al and ANF@M fibers

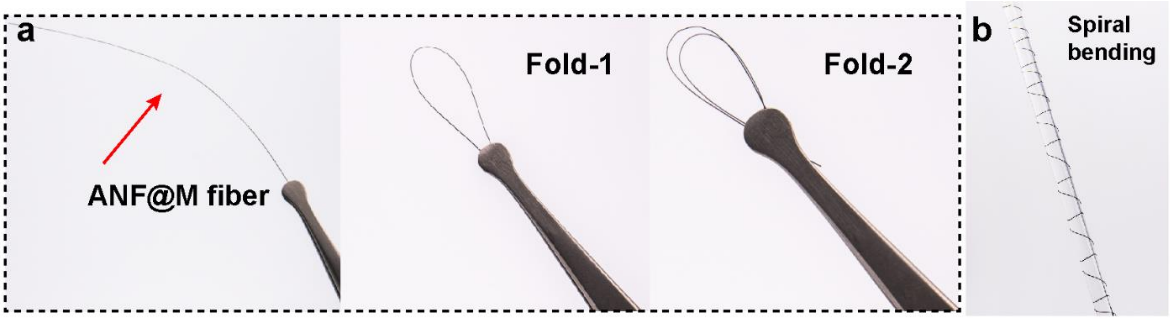

Fig. S21 Digital photos of **a** single ANF@M fiber and its folded counterparets, and **b** the fiber spirally wound on a glass rod

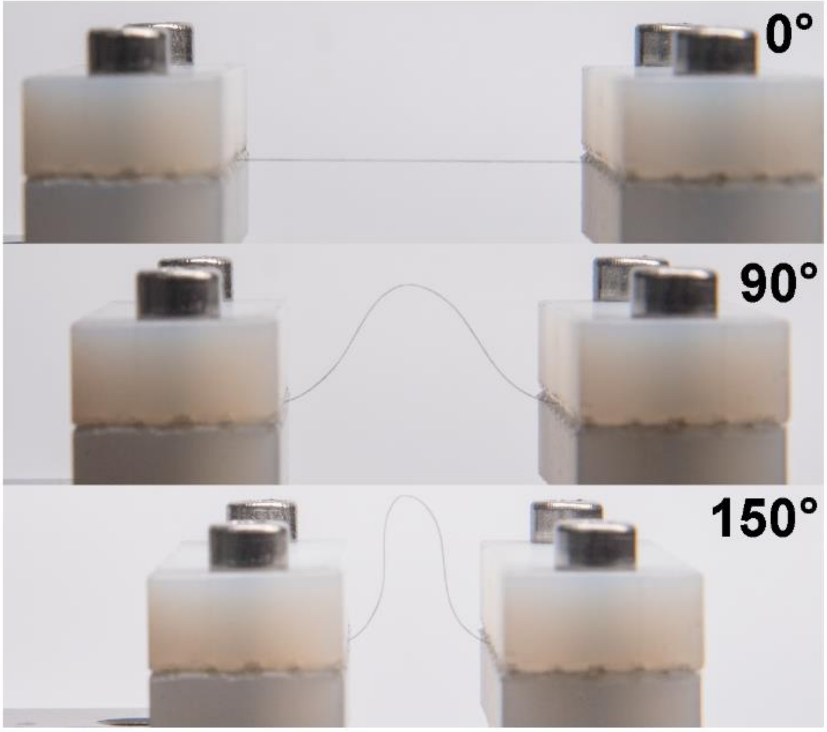

Fig. S22 Digital photos of different bending angles of ANF@M fibers

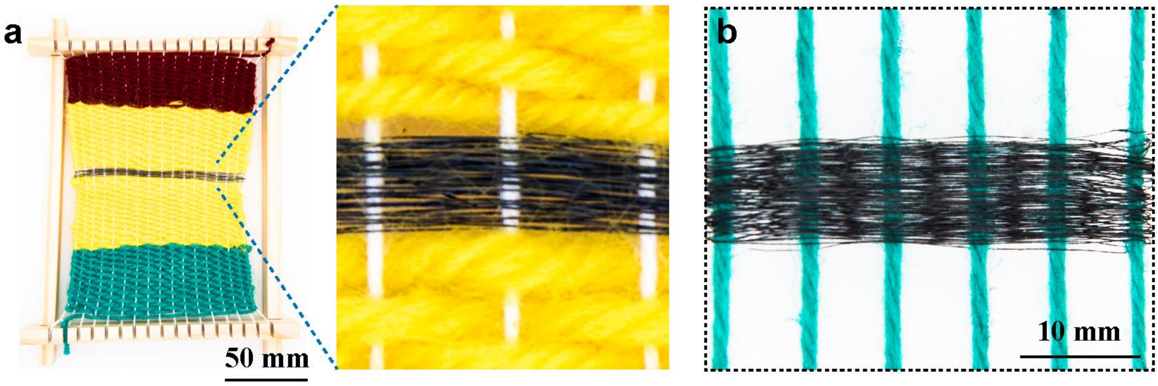

Fig. S23 Digital photos of **a** ANF@M fibers woven together by a simple manual loom and cotton thread, and **b** woven textile with the green cotton warp thread and the ANF@M fiber weft thread

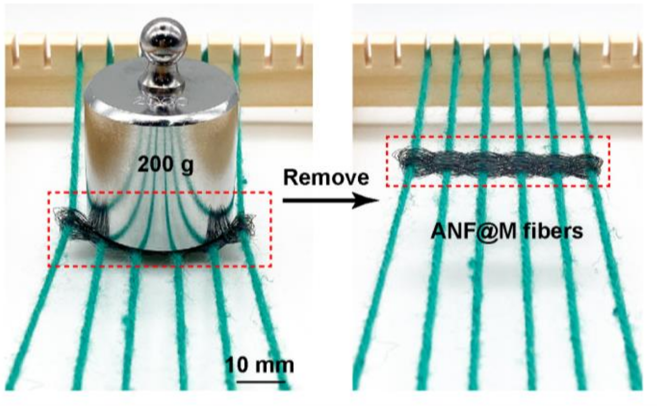

Fig. S24 The fibers textile can bear the pressure of 200 g, and there is no damage of the fibers after removing the weight

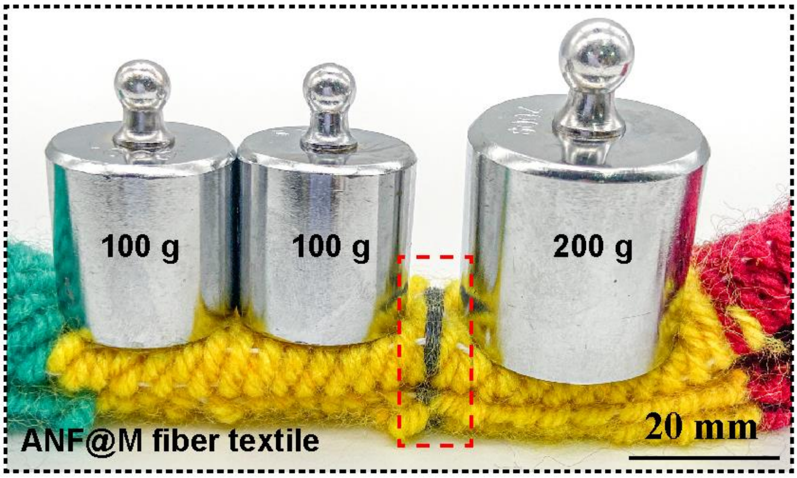

Fig. S25 Digital photo of an ANF@M fiber textile folded twice

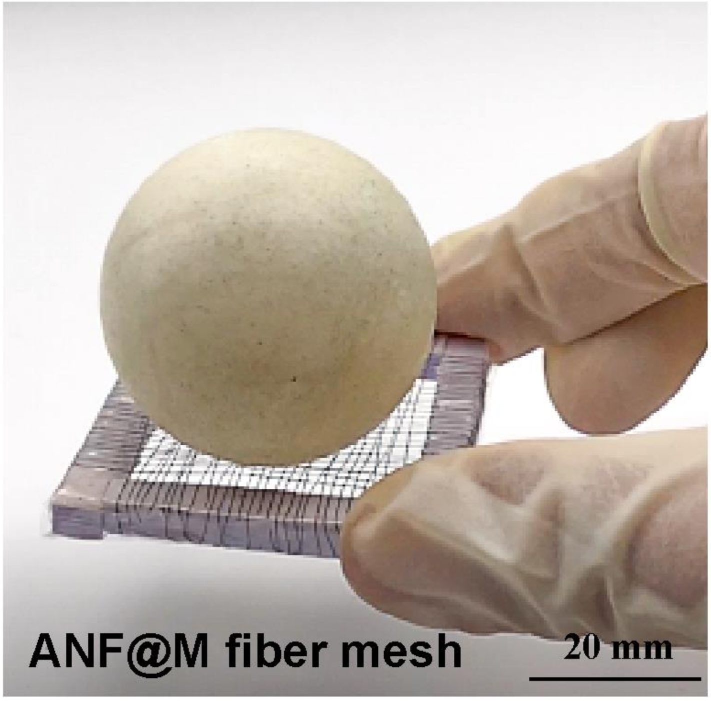

Fig. S26 Digital photo of ANF@M fibers woven into a net to bounce a table tennis

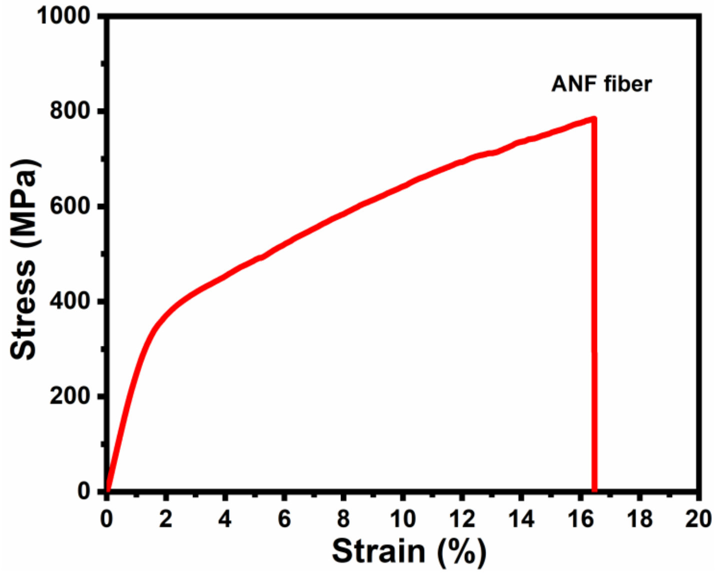

Fig. S27 Typical tensile stress–strain curve of the ANF fiber

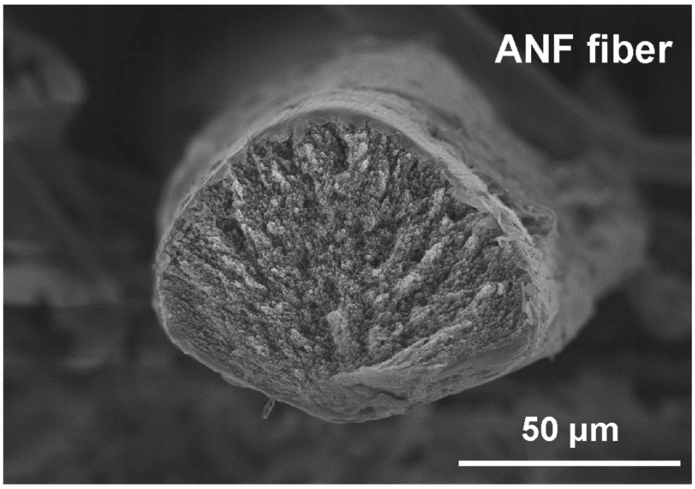

Fig. S28 Cross-sectional SEM image of ANF fiber

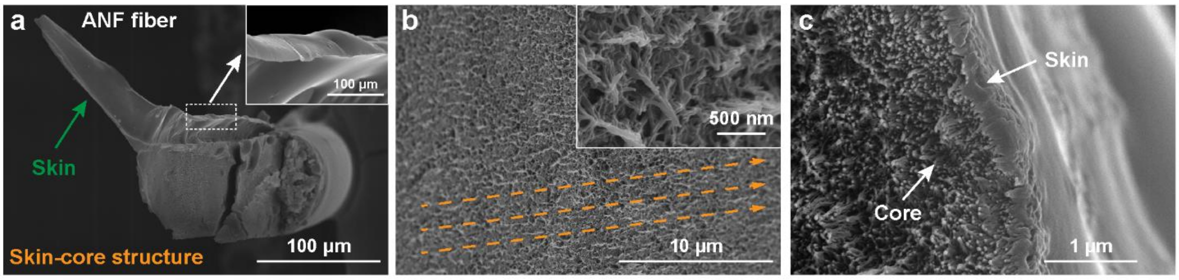

Fig. S29 a-c Cross-sectional SEM images of neate ANF fiber broken by tearing force under different magnifications

Different from the tabular core-shell ANF@M fiber, the nearly circular ANF fiber is composed of numerous closely stacked microfibers encapsulated by a smooth skin (**Fig. S29**). The obvious plastic deformation zone in the stress-strain curve of the neat ANF fiber indicates its supertough feature (**Fig. S27**). After fracturing, the microfibrils are highly stretched and orientated along the tensile direction (**Fig. S29b**), providing the ANF fiber with outstanding tensile strength and toughness.

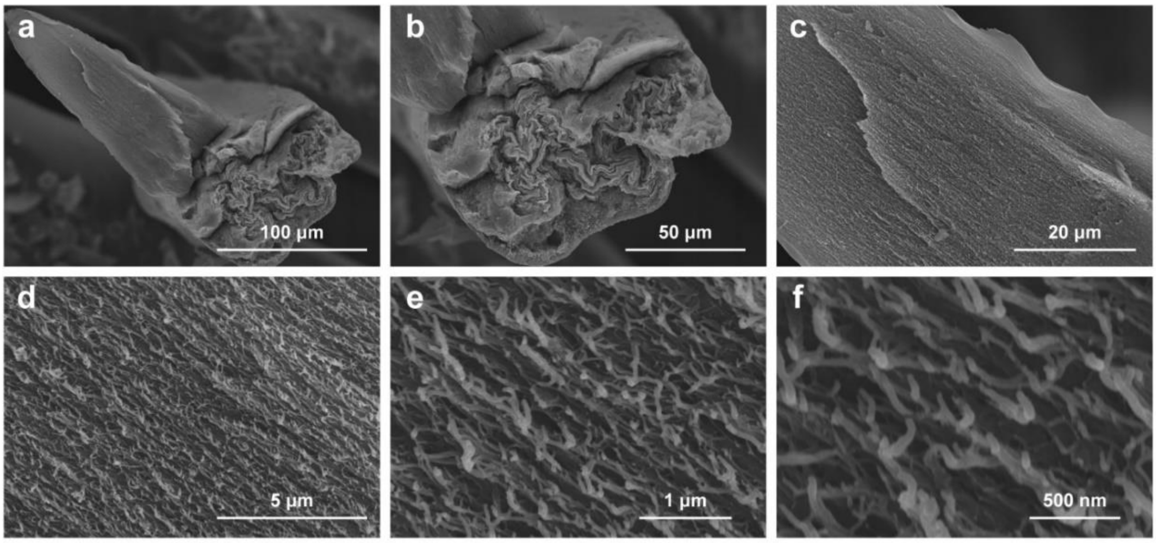

Fig. S30 a-f Cross-sectional SEM images of the ANF@M 16-1.1 fiber under different magnifications

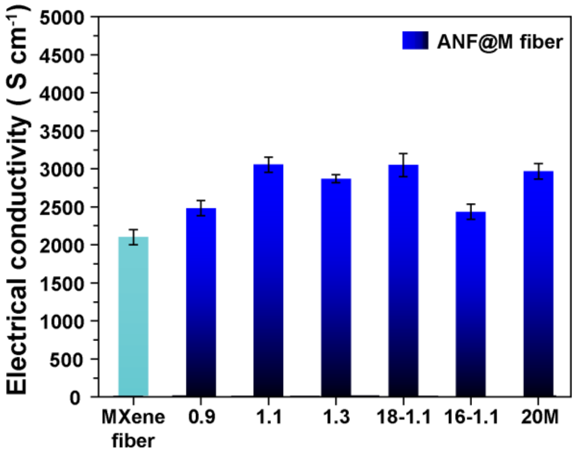

Fig. S31 Electrical conductivities of neat MXene fiber, and ANF@M fibers

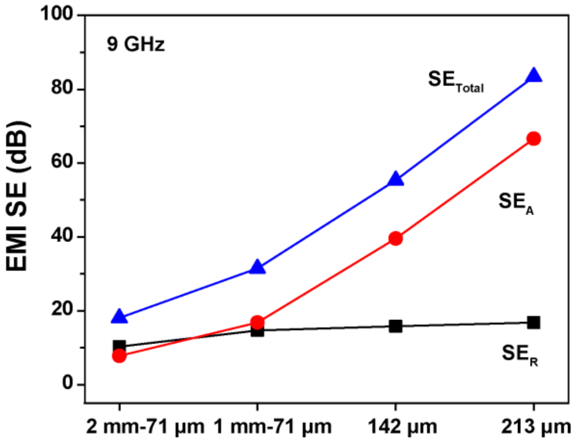

Fig. S32 Comparison of SE_{Total}, SE_A, and SE_R of the 17-1.1-50M textiles with different mesh sizes and thickneses at 9 GHz

The results of the reflection loss (SE_R), absorption loss (SE_A), and total shielding value (SE_{Total}) indicate the absorption-dominated EMI shielding mechanism (**Fig. S32**). When the mesh grid or the thickness of the conductive layer varies, the SE_R value is almost unchanged, while the SE_A increases linearly with the SE_{Total}, indicating that the decrease in the pore size or the increase

in the thickness of the conductive layer mainly affects the SE_A of the textile [S6]. The SE_R is mainly related to the electrical conductivity of the textile, while the SE_A is related to the structure of the textile. Small pores or multiple layers can effectively increase the SE_A, thereby increasing the EMI SE of the textile [S7, S8].

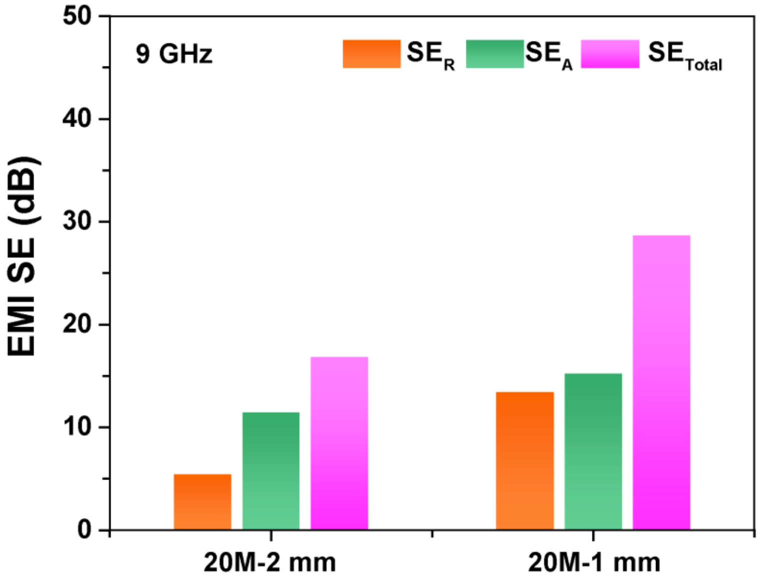

Fig. S33 Comparison of SE_{Total}, SE_A, and SE_R of the 17-1.1-20M textiles with different mesh sizes at 9 GHz

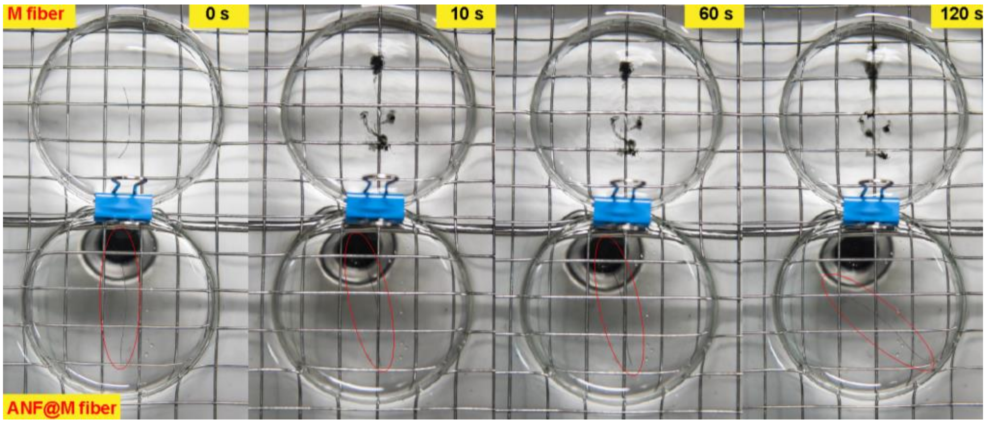

Fig. S34 Digital photos of neat MXene fiber and the ANF@M fiber after ultrasonic treatment for different periods

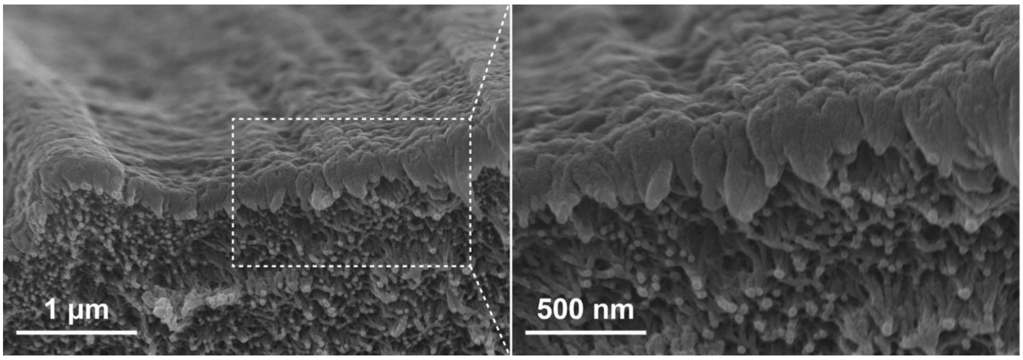

Fig. S35 Cross-sectional SEM images of the ANF@M fiber at different magnifications

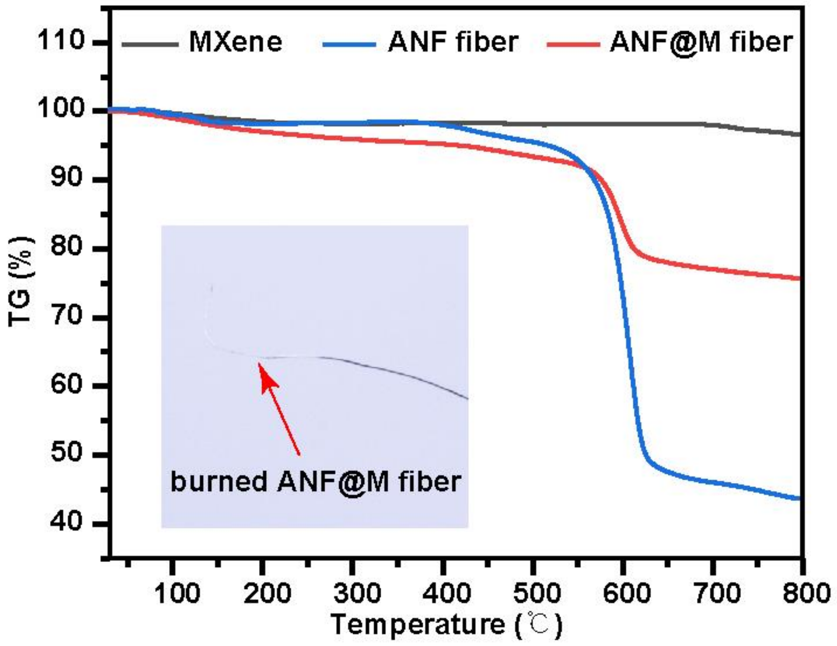

Fig. S36 Thermogravimetric analysis curves of neat MXene fiber, neat ANF fiber, and ANF@M fiber. The inset shows a treated ANF@M fiber by the flame of an alcohol lamp

Table S1 Comparison of densities, electrical conductivities, and mechanical properties of metal wires and ANF@M fibers

Samples	Density ($g\ cm^{-3}$)	Conductivity ($S\ m^{-1}$)	Tensile strength (MPa)	Toughness ($MJ\ m^{-3}$)
Silver wire	10.1	5.9×10^{7}	230.6	14.9
Copper wire	9.7	5.7×10^{7}	256.0	51.8
Aluminum wire	3.0	2.7×10^{7}	283.5	19.3
**ANF@M 17-1.1-50 M fiber**	3.2	3.1×10^{5}	380.1	34.9
**ANF@M 17-1.1-20M fiber**	2.6	3.0×10^{5}	502.9	48.1

Table S2 Comparison of the mechanical performances of ANF, MXene and ANF@M core-shell fibers

**Types**		**Tensile strength (MPa)**	**Young's modulus (GPa)**	**Elongation at break (%)**	**Toughness ($MJ\ m^{-3}$)**
Neat ANF fibers		784.7	27.0	16.5	92.4
Neat MXene fibers		59.9	22.1	0.6	0.2
ANF@M 17-1.1-50M fiber	ANF shell volume%	42.1	42.1	42.1	42.1
	MXene core volume%	57.9	57.9	57.9	57.9
	weighted value	365.0	24.2	7.3	39.2
	experimental value	380.1	29.0	11.7	34.9
ANF@M 17-1.1-20M fiber	ANF shell volume%	66.6	66.6	66.6	66.6
	MXene core volume%	33.4	33.4	33.4	33.4
	weighted value	542.6	25.4	11.2	61.6
	experimental value	502.9	26.6	13.1	48.1

Table S3 Diameter, shell thickness and mechanical performances of ANF@M core-shell fibers

Fibers	Diameter (μm)	Shell thickness (μm)	Tensile strength (MPa)	Elongation at break (%)	Modulus (GPa)	Toughness (MJ m^{-3})
17-0.9	79	11 ± 3	306.3	12.3	25.0	29.3
17-1.1	71	8 ± 2	380.1	11.7	29.0	34.9
17-1.3	66	7 ± 2	318.6	8.3	29.1	21.7
18-1.1	69	4 ± 1	230.5	3.4	26.5	6.2
16-1.1	92	23 ± 4	329.2	13.1	19.0	34.2
17-1.1-20M	68	15 ± 3	502.9	13.1	26.6	48.1

Table S4 Comparison of the mechanical properties of different MXene-based fibers

Fibers	Conductivity (S cm^{-1})	Strength (MPa)	Elongation (%)	Modulus (GPa)	Toughness (MJ m^{-3})	Refs.
MXene/rGO (90 wt.% MXene)	290	12.9	3.8			[S9]
MXene/rGO (15 wt.% MXene; uniaxial fiber)	58	132.5	2.9	11.3	2.7	[S10]
MXene/rGO (60 wt.% MXene)	743	110.7	7.6		3.8	[S11]
MXene@rGO (core)	24	290	1.5	30		[S12]
MXene/PEDOT (70 wt.% MXene)	1490	58.1	1.1	7.5		[S13]
MXene/ TOCNFs (50 wt.% MXene)	2	75.6	3.8	4.7		[S14]
MXene/nylon (90 wt.% MXene, 20 mg mL-1, 850 nm flakes)	1195	29.0	1.8	3.9		[S15]
Kevlar/ MXene (K1M2, 2wt% MXene)	0.17	20	3.0	1.7		[S16]
MXene/Aramid Fiber (5wt% MXene)	1025	104	7.9			[S17]
neat MXene	7750	40.5	1.7			[S4]
neat MXene	7713	63.9	0.22	29.6	0.09	[S18]
neat MXene	7200	118	1.8			[S19]
ANF@MXene-17-1.1-50M#	3053	380.1	11.7	29.0	34.9	This work
ANF@MXene-17-1.1-20M##	2969	502.9	13.1	26.6	48.1	

* rGO: reduced graphene oxide; PEDOT: poly(3,4-ethylenedioxythiophene); ANF: aramid nanofiber; TOCNFs: TEMPO (2,2,6,6-tetramethylpiperidine-1-oxylradi-cal)-mediated oxidized cellulose nanofibrils.

# The concentration of MXene solution is 50 mg mL^{-1};

The concentration of MXene solution is 20 mg mL^{-1}.

Video S1 The circular motion process.

Video S2 The ANF@M textile can withstand vigorous bending and kneading.

Video S3 A ball can be bounced on the net of the fibers.

Video S4 The ANF@M textile can bear the falling impact of a ball.

Supplementary References

[S1] G. Xin, W. Zhu, Y. Deng, J. Cheng, L.T. Zhang et al, Microfluidics-enabled orientation and microstructure control of macroscopic graphene fibres. Nat. Nanotechnol. **14**(2), 168-175 (2019). https://doi.org/10.1038/s41565-018-0330-9

[S2] S. Chen, W. Ma, Y. Cheng, Z. Weng, B. Sun et al., Scalable non-liquid-crystal spinning of locally aligned graphene fibers for high-performance wearable supercapacitors. Nano Energy **15**, 642-653 (2015). https://doi.org/10.1016/j.nanoen.2015.05.004

[S3] Z. Xu, Y. Liu, X. Zhao, L. Peng, H. Sun et al., Ultrastiff and strong graphene fibers via full-scale synergetic defect engineering. Adv. Mater. **28**(30), 6449–6456 (2016). https://doi.org/10.1002/adma.201506426

[S4] J. Zhang, S. Uzun, S. Seyedin, P.A. Lynch, B. Akuzum et al., Additive-free MXene liquid crystals and fibers. ACS Cent. Sci. **6**(2), 254-265 (2020). https://doi.org/10.1021/acscentsci.9b01217

[S5] W. Cao, L. Yang, X. Qi, Y. Hou, J. Zhu et al., Carbon nanotube wires sheathed by aramid nanofibers. Adv. Funct. Mater. **27**(34), 1701061 (2017). https://doi.org/10.1002/adfm.201701061

[S6] Z. Zeng, H. Jin, M. Chen, W. Li, L. Zhou et al., Lightweight and anisotropic porous MWCNT/WPU composites for ultrahigh performance electromagnetic interference shielding. Adv. Funct. Mater. **26**(2), 303-310 (2016). https://doi.org/10.1002/adfm.201503579

[S7] A. Iqbal, P. Sambyal, C.M. Koo, 2D MXenes for electromagnetic shielding: a review. Adv. Funct. Mater. **30**(47), 2000883 (2020). https://doi.org/10.1002/adfm.202000883

[S8] Z. Chen, C. Xu, C. Ma, W. Ren, H.M. Cheng, Lightweight and flexible graphene foam composites for high-performance electromagnetic interference shielding. Adv. Mater. **25**(9), 1296-1300 (2013). https://doi.org/10.1002/adma.201204196

[S9] Q. Yang, Z. Xu, B. Fang, T. Huang, S. Cai et al., MXene/graphene hybrid fibers for high performance flexible supercapacitors. J. Mater. Chem. A **5**(42), 22113-22119 (2017). https://doi.org/10.1039/c7ta07999k

[S10] S. Seyedin, E.R.S. Yanza, J.M. Razal, Knittable energy storing fiber with high volumetric performance made from predominantly MXene nanosheets. J. Mater. Chem. A **5**(46), 24076-24082 (2017). https://doi.org/10.1039/c7ta08355f

[S11] N. He, S. Patil, J. Qu, J. Liao, F. Zhao et al., Effects of electrolyte mediation and MXene size in fiber-shaped supercapacitors. ACS Appl. Energy Mater. **3**(3), 2949-2958 (2020). https://doi.org/10.1021/acsaem.0c00024

[S12] X. Chen, J. Jiang, G. Yang, C. Li, Y. Li, Bioinspired wood-like coaxial fibers based on MXene@graphene oxide with superior mechanical and electrical properties. Nanoscale **12**(41), 21325-21333 (2020). https://doi.org/10.1039/d0nr04928j

[S13] J. Zhang, S. Seyedin, S. Qin, Z. Wang, S. Moradi et al., Highly conductive $Ti_3C_2T_x$ MXene hybrid fibers for flexible and elastic fiber-shaped supercapacitors. Small **15**(8), 1804732 (2019). https://doi.org/10.1002/smll.201804732

[S14] W.T. Cao, C. Ma, D.S. Mao, J. Zhang, M.G. Ma et al., MXene-reinforced cellulose nanofibril inks for 3D-printed smart fibres and textiles. Adv. Funct. Mater. **29**(51), 1905898 (2019). https://doi.org/10.1002/adfm.201905898

[S15] A. Levitt, S. Seyedin, J. Zhang, X. Wang, J.M. Razal et al., Bath electrospinning of continuous and scalable multifunctional MXene-infiltrated nanoyarns. Small **16**(26), 2002158 (2020). https://doi.org/10.1002/smll.202002158

[S16] B. Cheng, P. Wu, Scalable fabrication of kevlar/$Ti_3C_2T_x$ MXene intelligent wearable fabrics with multiple sensory capabilities. ACS Nano **15**(5), 8676-8685 (2021). https://doi.org/10.1021/acsnano.1c00749

[S17] Q. Liu, A. Zhao, X. He, Q. Li, J. Sun et al., Full-temperature all-solid-state $Ti_3C_2T_x$/aramid fiber supercapacitor with optimal balance of capacitive performance and flexibility. Adv. Funct. Mater. **31**(22), 2010944 (2021). https://doi.org/10.1002/adfm.202010944

[S18] W. Eom, H. Shin, R.B. Ambade, S.H. Lee, K.H. Lee et al., Large-scale wet-spinning of highly electroconductive MXene fibers. Nat. Commun. **11**, 2825 (2020). https://doi.org/10.1038/s41467-020-16671-1

[S19] S. Li, Z. Fan, G. Wu, Y. Shao, Z. Xia et al., Assembly of nanofluidic MXene fibers with enhanced ionic transport and capacitive charge storage by flake orientation. ACS Nano **15**(4), 7821-7832 (2021). https://doi.org/10.1021/acsnano.1c02271
